# Harnessing nanozymes as next-generation antimicrobial agents: From mechanisms to therapeutic strategies

**DOI:** 10.1016/j.mtbio.2025.102499

**Published:** 2025-10-31

**Authors:** Shanshan Feng, Zhaoxun Wang, Yibin Zhang, Ling Mei, Zhenxing Wang

**Affiliations:** a Engineering Research Center for Pharmaceuticals and Equipments of Sichuan Province, Sichuan Industrial Institute of Antibiotics, School of Pharmacy, Chengdu University, Chengdu, 610106, China; b Traditional Chinese Medicine Hospital of Meishan, Meishan, 620020, China; c Hospital of Chengdu University of Traditional Chinese Medicine, Chengdu, 610072, China; d Infectious diseases Department, Traditional Chinese Medicine Hospital of Meishan, Meishan, 620020, China

**Keywords:** Nanozymes, Antimicrobial therapy, Reactive oxygen species, Biofilm, Catalytic mechanism

## Abstract

The escalating threat of antimicrobial resistance (AMR) and the limited efficacy of conventional antibiotics have intensified the search for alternative therapeutic strategies. Nanozymes, nanomaterials with enzyme-mimicking properties, have emerged as a promising class of antimicrobial agents due to their broad-spectrum activity, tunable catalytic functions, and resistance-independent mechanisms. This review provides a comprehensive overview of the recent advances in nanozyme-based antimicrobial applications, focusing on their classification, catalytic mechanisms, and multifaceted antimicrobial actions. We highlight the unique advantages of nanozymes, including their ability to generate reactive oxygen species (ROS), disrupt biofilms, modulate infection microenvironments, and synergize with other therapeutic modalities. Furthermore, we discuss their performance in various infection models, such as skin and soft tissue infections, oral infections, ocular infections, gastrointestinal infections, respiratory infections, urinary tract infections, systemic infections, diabetic wounds, chronic ulcers, and fungal infections. Finally, we address current challenges in clinical translation, including biosafety, dose control, and scalable production. This review underscores the transformative potential of nanozymes in combating drug-resistant pathogens and offers insights into future directions for their clinical development.

## Graphical abstract

**Figure:**
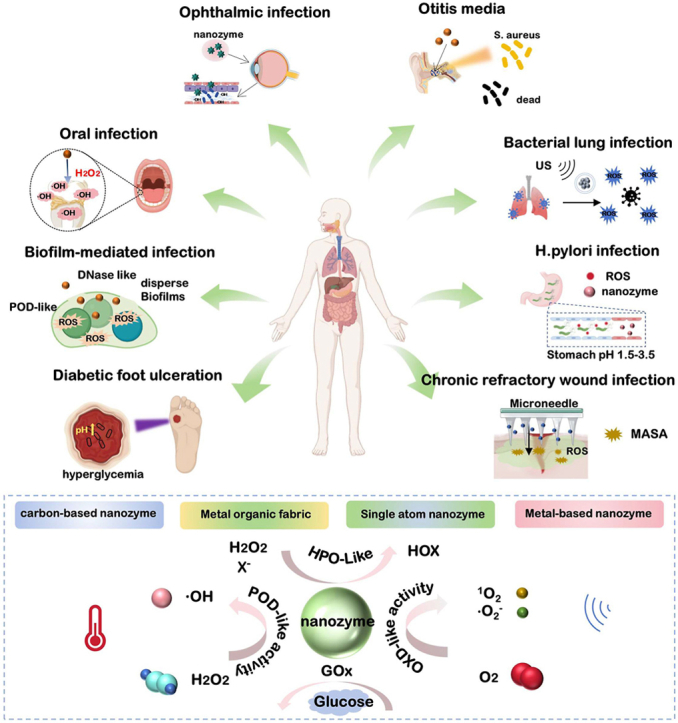

## 1 Introduction

### 1.1 Current situation of pathogenic infections

Pathogenic infections arise from a vast spectrum of microorganisms, including bacteria, fungi, viruses, and parasites, which collectively constitute a pervasive global threat to public health [1]. Among these etiological agents, bacterial and fungal pathogens are responsible for the majority of clinically significant infections. Bacterial infections remain highly prevalent; for instance, Streptococcus pneumoniae continues to cause a disproportionate burden of community-acquired pneumonia, particularly in developing countries [2]. These pathogens infiltrate the human host via multiple portals of entry—most notably the respiratory and gastrointestinal mucosae—thereby establishing systemic or localized infections. The advent of antibiotics—exemplified by tetracyclines, aminoglycosides, and macrolides—revolutionized infectious-disease management by targeting essential bacterial processes, including cell-wall synthesis, ribosomal protein translation, nucleic-acid replication, and key metabolic pathways [[3], [4], [5], [6], [7]]. However, the widespread use of antibiotics has gradually led to the emergence of bacterial resistance, posing a major challenge in treatment [8,9]. In recent years, there has been a notable increase in the incidence of fungal infections, particularly among patients with compromised immune function. Deep fungal infections, such as invasive fungal infections, have become severe, life-threatening conditions, and their diagnosis and treatment are challenging. Due to the structural differences in cell walls between fungi and bacteria, penicillins are ineffective against fungi. Only a limited number of antibiotics, such as polyene antibiotics (amphotericin B, nystatin) and azole drugs, are effective in treating fungal infections [10]. Nevertheless, with the use of antifungal drugs, fungal resistance is also gradually emerging. Therefore, the urgent need arises for the development of novel antimicrobial agents and strategies to address the issue of drug resistance (see Table 1, Table 2).

**Table 1:** Mechanisms of Nanozyme antimicrobial Activity and Their Advantages and Disadvantages.

Mechanism	Advantages	Potential limitations	Rf
ROS generation•OH/^1^O_2_via POD-/OXD-like catalysis	Broad-spectrum; multidrug-resistant (MDR)-proof.	Host oxidative injury if H_2_O_2_ or pH unfavorable	[[71], [72], [73], [74], [75]]
Physical membrane disruption Sharp/cationic surface pierces envelope	Instant kill; no biochemical escape	Morphology-dependent; corona masks edges.	[76]
Metal-ion release (Ag^+^, Cu^2+^, Zn^2+^) Slow leaching → thiol attack + ROS	Long-lasting; multi-target	Ion accumulation; emerging efflux pumps	[77,78]
Biofilm penetration & matrix degradation DNase-/protease-like + small size	Dismantles drug-tolerance fortress	Uneven depth in thick extracellular polymeric substance (EPS)	[[79], [80], [81], [82], [83]]
Photothermal/photodynamic synergy NIR heat (≥45 °C) or massive ROS burst	Single 5-min irradiation ≥5 log_10_ kill	Requires laser; risk of thermal by-stander damage	[73,84,85]

**Table 2:** Design strategies and therapeutic effects of nanozymes for different pathogenic infection disease models.

Disease model	Challenges for treatment	Design strategies for nanozymes	Core therapeutic roles	Rf
Subcutaneous/Deep Soft Tissue Infections	Hypoxia, poor penetration of physical therapies, difficult monitoring	Self-oxygen supplying; Microenvironment-responsive activation; Synergistic photothermal therapy	Broad-spectrum antibacterial activity, abscess elimination; Relieves hypoxia, scavenges ROS, promotes tissue regeneration.	[[96], [97], [98], [99]]
Oral Bacterial Infections	Complex microbiota, salivary clearance, biofilm formation	Adhesive systems (patches/hydrogels); *Anti*-biofilm nanozymes; pH-responsive activation	Targets caries/periodontal pathogens; Disrupts biofilms, reduces inflammation, maintains micro-ecology	[[100], [101], [102]]
Ophthalmic Bacterial Infections	Ocular barriers, tear clearance	Nanozyme-loaded microneedles for enhanced penetration & retention	Rapid bacterial clearance (e.g., *S. aureus*); Reduces inflammation, promotes corneal repair	[[103], [104], [105]]
Gastrointestinal Bacterial Infections	Acidic environment; non-specific killing; short retention time	pH-targeted activation; Surface modification for enhanced targeting; Nanozyme-probiotic synergy	Precisely inhibits pathogens *Helicobacter pylori* (*H. pylori*); Repairs mucosa, modulates flora balance	[[106], [107], [108], [109], [110]]
Chronic Refractory/Diabetic Wounds	Hyperglycemia, persistent inflammation, excessive oxidative stress	Hydrogel delivery; Multi-enzyme synergy; GOx-driven cascade reaction; pH-responsive ROS regulation	Highly effective sterilization (>95 %); Regulates redox homeostasis; Promotes angiogenesis & tissue repair	[[111], [112], [113], [114], [115], [116], [117], [118], [119], [120]]
fungal infection	Complex and robust cell wall (chitin, glucan); increasing drug resistance	Nanozyme-antifungal drug combination; Multi-mechanistic synergy	Disrupts cell wall/membrane; Induces oxidative stress & ferroptosis, effective clearance of diverse fungi	[22,[121], [122], [123], [124], [125]]
Biofilm infection	EPS barrier, hypoxic/acidic microenvironment, high drug resistance	DNase-like eDNA degradation; Photothermal synergy for penetration; QS interference	Effectively penetrates & disrupts biofilm structure; Eradicates embedded bacteria, alleviates inflammation	[[87], [88], [89], [90], [91], [92], [93]]

### 1.2 Antimicrobial potential of nanozymes: comparing the differential value of traditional antimicrobial agents

With the development of nanotechnology, the application of nanomedicine in the field of antimicrobial therapy is increasingly widespread [[11], [12], [13]]. Nano drug delivery technology can accurately deliver antimicrobial agents to the infection site, or use nano materials as carriers to realize the joint delivery of multiple antimicrobial agents [14]. Compared with the use of traditional antimicrobial agents, this new treatment method can achieve the purpose of reducing the dose and precise treatment, so as to effectively avoid the generation of drug resistance. In addition, some nanomaterials, such as Ag and Au, can also directly kill bacteria and play an antimicrobial role due to their inherent antimicrobial activity [15,16]. However, although these nanomaterials have many advantages in the field of antimicrobial, their biological safety and mechanism of action are not completely clear, which limits their further development and application to a certain extent. As a new antimicrobial agent, nanozymes have great potential, especially in dealing with serious bacterial drug resistance. Nanozymes are a class of nanomaterials with simulated natural enzyme activity. Since the discovery in 2007 that Fe_3_O_4_ has catalytic activity towards peroxidase (POD) substrates, research on nanozymes has continuously emerged [17]. Compared with traditional antibiotics, nanozymes exhibit strong broad-spectrum antimicrobial properties, low toxicity and economic effects.

#### 1.2.1 Key advantages of nanozymes in antimicrobial activity

**Unique antimicrobial mechanism and reduced resistance**: Traditional antibiotics eliminate bacteria by inhibiting bacterial metabolic pathways, such as cell wall synthesis and protein synthesis, but this approach readily induces resistance genes. In contrast, nanozymes exert synergistic effects through multiple mechanisms, including physical disruption (electrostatic adsorption leading to cell membrane lysis) [18], reactive oxygen species (ROS) oxidation (damaging DNA/proteins) [19,20], and hypohalous acid generation (inhibiting quorum sensing, QS). Consequently, it is challenging for bacteria to develop resistance through a single mutation. Notably, a study revealed that the polypeptide nanozyme Ni-IH-7 exhibits a clearance rate of over 90 % against various drug-resistant fungi, with no reported cases of resistance.

**Stability and environmental adaptability** Natural enzymes are often susceptible to inactivation by various environmental factors such as temperature and pH. For instance, horseradish peroxidase (HRP) is known to lose its activity at temperatures above 60 °C. In contrast, nanozymes, such as CeO_2_ and Fe_3_O_4_, exhibit remarkable stability and can maintain their catalytic activity under extreme conditions, including high temperatures and strong acidic or basic environments. This robustness makes nanozymes particularly suitable for complex infection microenvironments, such as the acidic milieu of diabetic wounds.

**Multifunctional synergy**: A single nanozyme can simultaneously mimic multiple enzymatic activities, such as POD, catalase (CAT), and superoxide dismutase (SOD). This capability enables the generation of ROS to eliminate bacteria and the modulation of oxidative stress to alleviate inflammation, thereby achieving dual functionality of "antimicrobial action and wound healing promotion". For instance, the degradable Cu PBG nanozyme reported in 2025 catalyzes the conversion of H_2_O_2_ to hydroxyl radicals (•OH) in acidic wound microenvironments for sterilization, while decomposing H_2_O_2_ into O_2_ in neutral microenvironments to facilitate repair. Beyond their enzyme-mimicking properties, nanozymes also serve as nanocarriers for the precise delivery of antimicrobial or anti-inflammatory drugs.

**Designability and targeting** The catalytic efficiency and selectivity of these nanozymes can be precisely optimized through strategic regulation of key parameters. This includes modulating the composition of diatomic active centers (Ce-O-Mn), controlling particle size (sub-100 nm dimensions enhance bacterial adsorption capacity), and implementing targeted surface modifications (such as hyaluronic acid functionalization for site-specific delivery to tumor/infection sites) [21,22]. Illustrative of this approach, recent work on silicon-based diatomic nanozymes demonstrated that enhanced catalytic activity via oxygen-bridge-mediated charge transfer. These constructs further mimic multi-enzyme functionalities and incorporate loaded plant polyphenols to augment therapeutic outcomes.

### 1.3 New breakthroughs in nanozyme research

The field of nanozyme research has achieved a series of exciting breakthroughs, bringing new possibilities in design and development, catalytic efficiency, intelligent response, and clinical translation.

**From Single-Atom to Dual-Atom Synergy:** The emergence of single-atom nanozymes marks a new phase of atomic-level precision design in nanozyme research. The key advance lies in anchoring metal active centers as isolated single atoms on a support, achieving nearly 100 % atomic utilization and fully uniform active sites. In contrast to the limited catalytic efficiency of traditional single-metal-site nanozymes, recent developments in dual single-atom nanozymes have enabled a leap in catalytic performance and antimicrobial efficacy through synergistic interactions between metals [23].

**Artificial intelligence (AI)-Assisted Nanozyme Design:** With the advancement of artificial intelligence, AI is driving a shift in nanozyme research from "trial-and-error" to "rational design". Traditional nanozyme development largely relied on empirical exploration and repetitive experimentation, which was time-consuming and inefficient. In recent years, the rapid progress of AI technology has brought transformative changes to nanozyme research, particularly in activity prediction, structural design, and synthesis optimization [24]. For instance, many recent studies have employed machine learning-assisted high-throughput screening strategies to accelerate the discovery of therapeutic nanozymes [25].

**From Simple** **"Sterilization" to Integrated** **"Diagnosis-Treatment-Healing":** As a novel type of antimicrobial agent, the translation of nanozymes into clinical applications is a fundamental challenge that needs to be addressed. Numerous studies have focused on designing nanozymes with clinical use in mind, aiming to leverage their antimicrobial advantages in practical settings. These materials are being developed as alternatives to conventional antimicrobials to combat the serious public health threat posed by bacterial resistance. Examples include antimicrobial coatings for medical devices and diagnostic-therapeutic technologies based on nanozymes for rapid detection of pathogenic microorganisms [[26], [27], [28]].

**Scheme 1:**
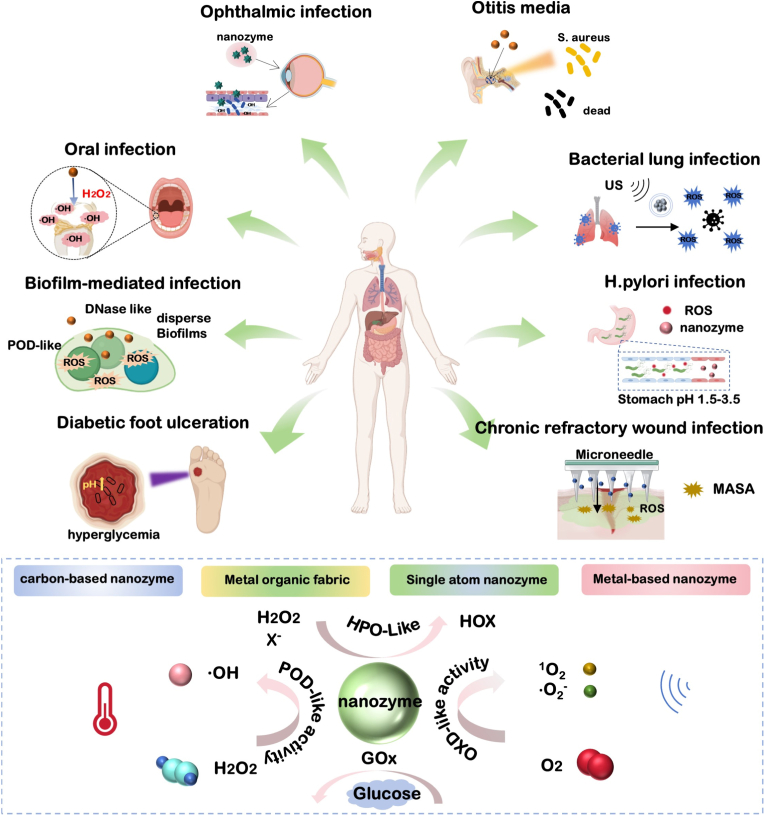
Schematic diagram of the application and mechanism of action of nanozymes in different pathogenic infectious diseases.

In summary, current antimicrobial therapy faces challenges such as the demand for broad-spectrum sterilization versus insufficient specificity, the spread of antimicrobial resistance lagging behind the development of new drugs, and the difficulty in balancing biosafety with efficacy. Nanozymes, by mimicking natural enzyme activities and leveraging the properties of nanomaterials, offer a unique pathway to address these contradictions. With their distinctive antimicrobial mechanisms, stability, and modifiability, nanozymes demonstrate significant potential and application prospects in the field of antimicrobials (Scheme 1). However, current research on nanozyme-based antimicrobials still has considerable limitations and shortcomings. Most studies tend to focus on constructing nanozyme systems to enhance their antimicrobial activity and investigating the underlying mechanisms. While such research is undeniably essential for the future development and application of nanozymes, their intended role as replacements for conventional antimicrobial agents demands compatibility with more complex clinical scenarios. The ability to adapt to the distinct pathological environments caused by different pathogenic microbe infections is a prerequisite for nanozymes to exert their antimicrobial effects and represents a critical step toward clinical translation. Therefore, the rational design and development of nanozymes based on diseases resulting from various pathogenic infections are equally important and directly influence their clinical applicability. So far, only a limited number of studies have designed and developed nanozymes based on disease models relevant to bacterial infections, such as basic wound infections and diabetic wound infections.

Accordingly, this review adopts the perspective of designing and developing nanozymes tailored to disease models of pathogenic infections. It summarizes current research on how to design nanozymes to adapt to different infection environments. The review begins by systematically outlining the classification of nanozymes, their enzymatic catalytic activities, and antimicrobial mechanisms, with a focused discussion on their application value in bacterial and other pathogenic infections. This includes: designing treatment strategies based on different pathogen infection models, enhancing nanozyme stability in varied pathogenic environments through structural modifications, and ultimately achieving synergistic antimicrobial and therapeutic outcomes. Finally, the article provides an in-depth analysis of key technological advances in the clinical application of nanozymes, as well as the challenges and future directions for their practical use. We hope this review will offer new insights and practical guidance for the clinical translation of nanozymes.

## 2 Classification and catalytic activity of nanozymes

### 2.1 Classification of nanozymes

Nanozymes are primarily classified by constituent elements and catalytic mechanisms. Based on composition, they comprise metal oxide, precious metal, and carbon-based types. Metal oxide nanozymes typically involve transition metal nanoparticles that exhibit redox activity through valence state changes and electron transfer during substrate interaction. Precious metal and carbon-based nanozymes exert catalytic function via adsorption, activation, and surface electron transfer.

#### 2.1.1 Metal nanozymes

##### 2.1.1.1 Noble metal nanozymes

Precious metal nanozymes (Au, Ag, Pt) demonstrate significant antimicrobial potential, leveraging their distinct physicochemical properties and enzyme-mimetic activities. These nanozymes primarily catalyze H_2_O_2_ decomposition through POD-like activity, generating ROS that disrupt bacterial structures [16,29] precious metal nanozymes exhibit photothermal effects upon photoexcitation, acting synergistically with ROS to enhance bactericidal efficacy. Du et al. developed NA-Ag@Pt nanozymes with POD-like activity that efficiently catalyze ROS generation from H_2_O_2_ [30], achieving 100 % and 85 % antimicrobial efficiency against *Escherichia coli* (*E. coli*) and *Staphylococcus aureus* (*S. aureus*), respectively. The team further engineered a Pb^2+^ sensor by incorporating Pb^2+^-specific aptamers to enhance electron transfer efficiency, demonstrating multifunctional application potential. Zhao et al. synthesized inulin-stabilized gold nanoparticles (IN@AuNPs) through in-situ reduction of Au^3+^ by inulin hydroxyl groups. As an antimicrobial agent, IN@AuNPs catalyze ROS generation, exhibiting efficacy against both Gram-negative (*E. coli*) and Gram-positive (*S. aureus*) bacteria. Surface hydroxyl groups enhance bacterial adhesion, reducing ROS diffusion distance and enabling complete bacterial eradication within 1 h [31]. Researchers designed AgPd alloy nanocages that efficiently catalyze in situ generation of surface-adsorbed ROS. These nanocages achieve effective elimination of bacteria (including drug-resistant strains) while maintaining biocompatibility with mammalian cells. This selective antimicrobial efficacy arises from two synergistic mechanisms: (1) surface-adsorbed ROS remain localized versus freely diffusible states, and (2) mammalian cells internalize nanoparticles via endocytosis, detoxifying surface-bound ROS—a process absent in bacteria [32]. Despite higher synthesis complexity and cost, precious metal nanozymes offer superior corrosion resistance and catalytic activity.

##### 2.1.1.2 Metal oxide/sulfide nanozyme

Metal oxide (e.g., Fe_3_O_4_, CeO_2_, ZnO) and sulfide (MoS_2_) nanozymes exert antimicrobial effects via synergistic mechanisms: (1) mimicking oxidoreductase activities (POD-/oxidase, OXD-like) to catalyze ROS generation; (2) releasing metal ions to disrupt bacterial metabolism; (3) enhancing enzymatic activity through photothermal effects. Bai et al. developed heterostructured ZnO nanorods@grapdiyne nanosheets (ZnO@GDY NRs) combining POD-like activity with piezoelectric properties, enabling ultrasound-triggered H_2_O_2_ decomposition into ROS. This system achieves near-complete eradication (>99.9 %) of multidrug-resistant pathogens including methicillin-resistant *Staphylococcus aureus* (MRSA) and *Pseudomonas aeruginosa* [33]. Separately, Li et al. engineered macrophage membrane-coated mesoporous CeO_2_-TCPP nanozymes, where: (i) pathogen-activated membrane encapsulation enhances bacterial targeting; (ii) CeO_2_ acts as both TCPP carrier and POD-mimic; (iii) synergistic ROS generation between CeO_2_ and photosensitizer TCPP under photodynamic conditions enables efficient biofilm elimination and accelerated wound healing [34].

##### 2.1.1.3 Metal organic framework nanozyme

Metal-organic frameworks (MOFs), porous crystalline coordination polymers formed between metal ions/clusters and organic ligands, exhibit high surface areas, tunable pore dimensions, and abundant catalytic metal sites. These properties confer exceptional nanozyme activity, establishing MOFs as emerging antimicrobial platforms with distinctive advantages [[35], [36], [37]]. MOF-based nanozymes function through three primary mechanisms: (1) POD-like activity catalyzing H_2_O_2_ conversion to ROS; (2) serving as carriers for antimicrobial agents (metallic nanoparticles, enzymes); (3) transformation into highly active single-atom nanozymes. Wang et al. engineered an acid-enhanced dual-mode antimicrobial nanozyme from ZIF-8, leveraging: (1) Zn^2+^ release for direct bactericidal activity; (2) Glucose Oxidase (GOx)/Au nanoparticle cascades where GOx generates H_2_O_2_ from glucose, while Au NPs exhibit POD-like activity to produce ROS; (3) gluconic acid byproducts that acidify the microenvironment to optimize catalysis. This synergy achieved MIC values of 4 μg/mL (*S. aureus*) and 8 μg/mL (*E. coli*) [38], Niu et al. synthesized Fe-N-C single-atom nanozymes (SANs) via MOF-derived carbon supporting atomically dispersed Fe-N_x_ sites. The Fe-N_x_/porous carbon synergy demonstrated exceptional POD-like activity (57.76 U·mg^−1^ specific activity) [39]. MOF nanozymes show significant potential for low-dose sterilization and resistance mitigation through structural tunability and multifunctionality, though stability and scalable production require further development.

##### 2.1.1.4 Metal single atom nanozyme

Metal single-atom nanozymes achieve near-complete atomic utilization and exceptional catalytic activity through isolated metal atoms anchored on carriers (carbon-based materials, metal oxides). Their antimicrobial properties arise from three mechanisms: (1) atomic-level active sites enabling enhanced POD-like activity and efficient ROS generation; (2) photothermal/photodynamic effects; (3) depletion of bacterial antioxidants (glutathione, GSH) to disrupt cellular redox homeostasis. Li et al. enhanced Fe-N_4_ catalytic activity by transforming 2D structures into 3D active centers through sulfur-oxide functionalization. This modification improved substrate adsorption and H_2_O desorption, achieving a specific activity of 119.77 U·mg^−1^—6.8-fold higher than conventional FeN_4_C Signle Atom nanozymes (SAzymes) [40]. Wang et al. engineered Cu single-atom sites on N-doped porous carbon (Cu SASs/NPC) nanozymes exhibiting triple functionality: (1) POD-like activity generating •OH from H_2_O_2_; (2) NIR photothermal enhancement of catalytic activity with additional •OH production; (3) glutathione peroxidase (GPx)-mimetic activity depleting bacterial GSH. This synergistic mechanism achieved near-complete bacterial eradication. Metal single-atom nanozymes represent a promising strategy for efficient, low-resistance antimicrobial therapy through atomic-level design and multimodal synergy. However, carrier-active site interactions and long-term biosafety require further investigation [41].

#### 2.1.2 Carbon based nanozymes

Carbon-based nanozymes are synthetic nanomaterials centered on carbon matrices, exhibiting tunable catalytic activity, robust regenerability, and exceptional stability. The multiple electron hybridization configurations (sp^2^/sp^3^) of carbon impart versatile structural properties and broad functionalization capacity. Classified by composition and structure, they comprise: (1) Intrinsic carbon-based nanozymes (graphene, carbon nanotubes, carbon quantum dots); (2) Composite carbon-based nanozymes (carbon-metal oxide heterostructures); (3) Heteroatom-doped carbon-based nanozymes (N/S/metal-doped carbons) [42] materials include fullerenes, graphene oxide (GO), and carbon aerogels. Their distinctive electronic structures enable precise modulation of enzyme-mimetic activities through surface engineering or heteroatom doping. Carbon-based nanozymes exert antimicrobial effects primarily through POD-like catalytic ROS generation or synergistic photothermal bactericidal activity. Shen et al. developed glucose-responsive photothermal nanozymes comprising GOx and Fe-doped carbon dots (Fe@CDs) integrated within mesoporous polydopamine (MPDA). This system sequentially: (1) generates H_2_O_2_ via glucose oxidation by GOx; (2) decomposes H_2_O_2_ into •OH through Fe@CD-enhanced POD-like activity; while (3) MPDA's photothermal effect disrupts cellular membranes. This cascade overcomes the H_2_O_2_ insufficiency limitation in conventional photodynamic and chemodynamic therapies (PDT,CDT) [43]. Separately, Dai et al. synthesized carbon nitride quantum dots (CNQDs) with high nitrogen-vacancy (N-v) concentrations via propylene glycol-assisted ultrasonication. N-vacancies expand π-electron delocalization to enhance POD-like activity while enabling membrane penetration for targeted •OH delivery. CNQDs demonstrated >99 % eradication against Gram-negative (*E. coli*), Gram-positive (*S. aureus*, B. subtilis), and fungal (F. graminearum) pathogens [44]. The inherent biocompatibility and biodegradability of carbon matrices further facilitate infected wound repair. With tunable electronic structures, multimodal antibacterial mechanisms (ROS/photothermal/biofilm penetration), and favorable biosafety profiles, carbon-based nanozymes show significant clinical potential. However, catalytic adaptability in complex biological microenvironments, scalable manufacturing, and chronic toxicity require systematic investigation to enable clinical translation.

### 2.2 Catalytic mechanism of nanozymes

#### 2.2.1 POD like activity

**Fig. 1:**
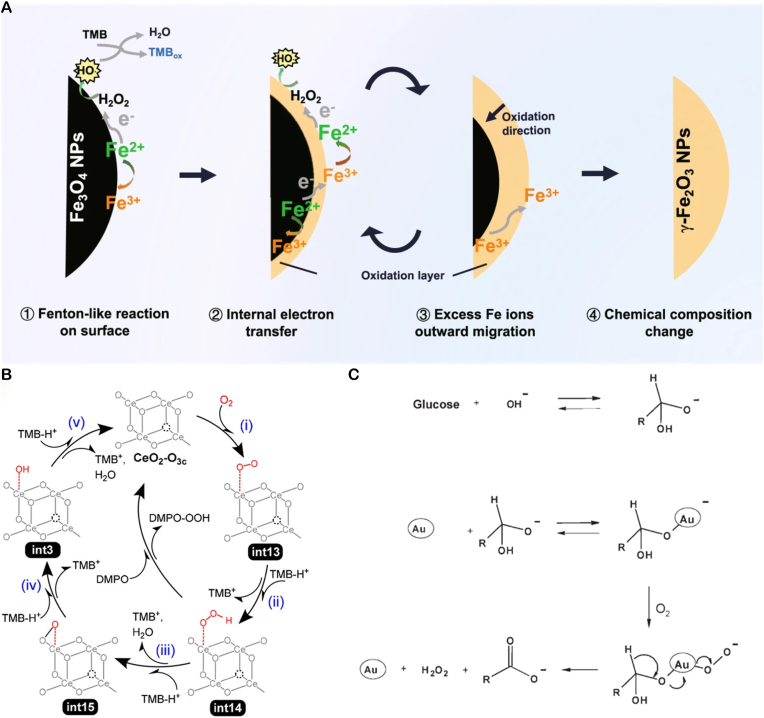
Schematic illustration of the catalytic mechanism of nanoparticles: (**A**) Proposed catalytic mechanism of Fe_3_O_4_ nanoparticles' POD-like activity. Copyright 2025 Springer Nature Limited. (**B**) The proposed mechanism for the OXD-like activity of nanoceria. Copyright 2021 American Chemical Society. (**C**) The proposed mechanism for the GOx-like activity of Au NPS. Copyright 2006 Wiley-VCH.

POD-like activity denotes the catalytic decomposition of hydrogen peroxide (H_2_O_2_) by nanozymes to generate highly reactive hydroxyl radicals (•OH). These radicals mediate antimicrobial effects through oxidative damage to bacterial membranes, proteins, nucleic acids, and other macromolecular structures. Numerous metal oxide nanozymes, including manganese dioxide and iron oxide variants, exhibit such POD-mimetic activity. Exemplified by iron-based nanozymes, early studies established that Fe_3_O_4_ nanozymes follow Fenton-type reaction mechanisms. Under acidic conditions, only surface-exposed ferrous (Fe^2+^) ions participate in catalysis, constraining enzymatic activity to interfacial regions [17]. Recent mechanistic insights reveal four interconnected pathways governing Fe_3_O_4_ POD-mimetic catalysis [45]: (Ⅰ) Surface Fenton-like reactions (Ⅱ) Internal electron transfer via Fe^2+^-O-Fe^3+^ chains, regenerating surface Fe^2+^ through adjacent subsurface ferrous ions (Ⅲ) Cation migration where excess Fe^3+^ diffuses toward the surface to maintain charge neutrality during subsurface Fe^2+^ oxidation, generating cationic vacancies (Ⅳ) Compositional evolution wherein progressive surface-to-core oxidation drives phase transformation to γ-Fe_2_O_3_ nanoparticles (Fig. 1A) [46].

#### 2.2.2 OXD like activity

Nanoparticles with nanozyme properties can directly catalyze the oxidation of O_2_ to generate ROS such as O_2_^−^, which damage bacterial cell membranes and intracellular components, thereby inhibiting bacterial growth or causing bacterial death. Certain metal-based nanoparticles, like gold and silver nanoparticles, possess oxidase-like activity. These nanoparticles can exert antimicrobial effects using atmospheric oxygen without the need for additional hydrogen peroxide. Wang et al. employed density functional theory (DFT) calculations to elucidate the atomic mechanism of the OXD-like activity of nanoceria. In this process, O_2_ first adsorbs onto the cerium (Ce) sites on the nanoceria surface to form intermediate int13. It is then reduced to form the hydrogen peroxide (HO_2_) intermediate int14, which is further reduced to the oxygen (O) intermediate int15. In int15, the oxygen adsorbate tends to remain at the surface oxygen sites and can be reduced to water (H_2_O) through several subsequent reactions (Fig. 1B) [47].

#### 2.2.3 Halogen peroxidase activity

Halogen peroxidase like enzymes (such as chloroperoxide and bromoperoxidase) catalyze the generation of highly oxidizing substances such as hypochlorous acid (HOCl) or hypobromous acid (HOBr) from H_2_O_2_. These substances can also damage the integrity of bacterial cell membranes, alter their permeability, and ultimately lead to bacterial death. Hypochloric acid or hypobromic acid can not only directly kill bacteria, but also specifically quench bacterial QS molecules and inhibit the formation of biofilms [48].

#### 2.2.4 Glucose oxidase like activity

GOx specifically catalyzes the oxidation of β-D-glucose, consuming environmental oxygen and continuously producing two key antimicrobial substances: gluconic acid and hydrogen peroxide. The accumulation of gluconic acid significantly lowers the local pH, creating an acidic environment that is unfavorable for the survival of most bacteria, directly inhibiting their growth. The produced H_2_O_2_ itself possesses antimicrobial properties, and more importantly, it can serve as a "fuel" for other catalysts. When combined with nanozymes exhibiting POD-like activity (such as Fe_3_O_4_), it can initiate efficient CDT, generating a large amount of highly toxic hydroxyl radicals in situ, thereby delivering a devastating blow to bacteria. Gold nanoparticles can exhibit catalytic activity under certain specific conditions. The gold atoms on its surface can interact with oxygen molecules, making it easier for oxygen molecules to accept electrons and be reduced. At the same time, certain functional groups in glucose molecules can provide electrons to gold nanoparticles. During this electron transfer process, glucose molecules are oxidized to gluconic acid and produce hydrogen peroxide (Fig. 1C) [49]. Unlike natural GOx, gold nanoparticles do not have a specific substrate recognition cavity, expanding the applicability of substrates and catalyzing different types of monosaccharide oxidation processes.

#### 2.2.5 SOD like activity

SOD is a crucial antioxidant enzyme in living organisms, and its role in anti-infection therapy is more sophisticated and complex. During infection, immune cells generate large amounts of ROS (including O_2_•^-^) to kill bacteria, but excessive ROS can also damage healthy tissues. Exogenous supplementation of SOD can clear the excess O_2_•^-^, alleviating inflammatory damage and promoting tissue repair. Constructing a cascade reaction represents one of its most ingenious potential applications. The H_2_O_2_ produced by SOD from O_2_•^-^ serves as a substrate for GOx or other POD. A GOx/SOD synergistic system can be designed: GOx consumes glucose and oxygen to produce acid and H_2_O_2_ for sterilization, while potentially generating a small amount of O_2_•^-^; meanwhile, SOD recycles these O_2_•^-^, converting them into more H_2_O_2_. This significantly enhances the overall ROS production efficiency, forming an intensified antimicrobial cycle. SOD activity depends on its metal cofactors (such as copper, zinc, manganese). Many nanozymes, such as CeO_2_, have metal centers that can quickly switch between different valence states, similar to the metal cofactors in natural SOD, such as copper and zinc. For example, Ce in CeO_2_ can be rapidly converted between Ce^3+^ and Ce^4+^, and this redox cycle enables CeO_2_ to catalyze the dismutation reaction of O_2_^−^ [50,51].

## 3 Structural regulation and nanoengineering: activity optimization strategies based on structure activity relationships

### 3.1 Lattice and defect engineering: atomic level control and optimization of electronic structure

The catalytic activity of nanozymes is highly dependent on their intrinsic electronic structure and surface properties. Through precise "crystal engineering" strategies, such as modulating lattice parameters and introducing defects, their electron distribution can be effectively optimized, thereby significantly enhancing their ability to adsorb and activate substrates. These strategies primarily include the following two approaches:

**Lattice Engineering: Modulating Electronic Structure to Lower Reaction Energy Barriers** The core of lattice engineering lies in directly modulating the electronic structure of the material, particularly the d-band electronic states of the catalytic active sites, by altering the interatomic distances. Lattice expansion is a typical strategy. When the lattice spacing is increased, the overlap of electron orbitals between metal atoms decreases, leading to a shift in the position of the d-band center. An upward shift of the d-band center can enhance the electron interaction between the nanozyme and the reaction substrate, thereby optimizing the adsorption strength of reaction intermediates and effectively lowering the energy barrier of the transition state throughout the catalytic process, accelerating the reaction kinetics. For instance, Li's team demonstrated that after artificially expanding the lattice spacing of ruthenium (Ru) nanozymes from 0.23 nm to 0.25 nm, the optimized d-band electronic structure resulted in a remarkable 3-fold increase in the rate of ROS generation under electrical stimulation. Parallel lattice distortion approaches applied to copper nanozymes similarly enhanced POD-mimetic activity, enabling efficient eradication of drug-resistant bacterial pathogens [52].

**Defect Engineering: Creating Highly Active Sites and Synergistic Effects** Defect engineering involves actively introducing "imperfect" sites (such as vacancies or dopants) into the ordered crystal structure to break chemical inertness and create a large number of highly active sites with unsaturated coordination. Among these, the introduction of oxygen vacancies is very common. Taking the classic Fe_3_O_4_ nanozyme as an example, creating oxygen vacancies in its crystal structure leads to a redistribution of local electron density, often manifested as a significant increase in the Fe^2+^/Fe^3+^ ratio. These electron-rich Fe^2+^ sites can more efficiently catalyze the Fenton reaction with hydrogen peroxide, generating highly cytotoxic hydroxyl radicals. Studies have reported that this method can enhance the POD-like activity of Fe_3_O_4_ by up to 4-fold [53].

Furthermore, metal doping is another highly effective defect engineering strategy. Doping noble metal atoms (e.g., Au, Pt) into the lattice of base nanozymes (e.g., Fe, Co, Cu-based) can form bimetallic alloy structures. The electronic and geometric effects between the different metal atoms can create a powerful synergistic effect, not only improving catalytic efficiency but also enhancing reaction selectivity, enabling the precise promotion of specific catalytic pathways [54,55].

**Single-Atom Site Engineering** The single-atom site design has shown great potential in enhancing the catalytic activities of nanozymes. The Fe-N_5_ SAzyme, a five-coordinated single-atom iron nanozyme, was synthesized via a high-temperature pyrolysis strategy. Its POD-like activity was remarkably enhanced by 3.45 × 10^5^ times compared to traditional Fe_3_O_4_. Density functional theory calculations revealed that the axial fifth nitrogen ligand in Fe-N_5_ SAzyme significantly improves the adsorption and activation of H_2_O_2_, thus boosting its catalytic performance [56]. In another example, the sulfur-engineered single-atom copper nanozyme (Cu-N/S-C) was developed. It contains a unique Cu-N_1_S_2_ site, which endows the nanozyme with both POD-like and CAT-like activities. This dual-enzymatic-like activity allows Cu-N/S-C to perform multiple catalytic functions in a single nanozyme platform. In addition, researchers used Pickering emulsification method to embed Pd single atom nanozyme into mesoporous selenium (MSe) nanozyme, constructing a self targeting enzyme with autonomous targeting ability Pd@MSe-TPP Nano motor for the treatment of rheumatoid arthritis [57].

### 3.2 Heterogeneous structure design: interface collaboration and Cascade Catalysis

By constructing heterogeneous interfaces and utilizing electron transfer or spatial confinement effects between different materials, multiple enzyme cascade reactions or microenvironment regulation can be achieved.

**Bimetallic heterostructure:** Cai's team synthesized Au Pt heterostructure aerogels, whose interface grain boundaries and strain effects significantly enhanced the glucose cascade reaction activity (2.8 times higher than the alloy structure activity). Sub nanometer level 3D imaging shows that interface distortion promotes the desorption and adsorption equilibrium of reaction intermediates (•OH, H_2_O_2_) [58].

**Zn Co oxide core-shell structure:** The core-shell heterojunction interface forms a charge gradient, accelerates electron transfer, and achieves targeted generation of ROS in an acidic microenvironment for tumor specific therapy.

**MOF - Restricted nanozymes:** Confinement of Polyacrylic Acid (PAA) within MOF pores creates a localized acidic microenvironment. This allows the nanozyme to maintain high activity at physiological pH 7.4, enhancing cascade reaction efficiency by 4-fold [59]. ZIF-8-Derived Single- Atom Zinc nanozyme: The Zn-N-C single-atom catalyst (PMCS), derived from ZIF-8, coordinates unsaturated Zn-N_4_ sites. This promotes uniform H_2_O_2_ cleavage into •OH, achieving a 99.87 % inhibition rate against P aeruginosa [60].

### 3.3 Size and morphology control: optimization of exposed active sites and specific surface area

The catalytic activity of nanozymes is positively correlated with their specific surface area and the degree of exposure of active sites. Nanozymes with smaller particle sizes have a higher proportion of surface atoms, which increases the likelihood of active sites (such as Fe^2+^) coming into contact with substrates, thereby accelerating reaction kinetics. For instance, Fe_3_O_4_ nanoparticles with a diameter of less than 5 nm exhibit a tenfold increase in POD-like activity compared to their micrometer-sized counterparts. This enhancement is attributed to the nanoparticles' high specific surface area and density of dangling bonds.

Heterosexual Morphology Design: In terms of heterosexual morphology design, two-dimensional MoS_2_ nanosheets demonstrate superior oxidase - like activity compared to bulk materials. This is due to the abundant sulfur vacancies at their edges [61]. Similarly, one-dimensional CuO nanowires enhance ROS generation efficiency through axial electron transfer pathways [62].

Porous Structure: Regarding porous structures, Au nanozymes loaded on mesoporous silica exhibit a threefold increase in catalytic efficiency. This is achieved through the pore confinement effect, which enriches substrates around the nanozymes.

### 3.4 Composite system construction: multi-functional collaboration and stability enhancement

The stability of nanozymes can be enhanced and their application scenarios expanded by combining them with other functional materials.

**The Photothermal Catalytic Synergistic System** Xu's team designed liposomes co-loaded with VOx nanozymes and photosensitizer Ce6 (VC@Lipo). In the tumor microenvironment, the synergy between CDT and PDT increases ROS generation by five times [63].

**Ferritin Nanocages** Fan's team used ferritin cavities to load single-atom nanozymes. Through targeted receptor-mediated endocytosis, the nanozymes are specifically enriched at the tumor site, reducing systemic toxicity.

## 4 Multidimensional sterilization and microenvironment regulation of nanozymes against bacterial resistance—from antimicrobial pathways to resistance circumvention

### 4.1 The emergence of bacterial resistance and the development of novel antimicrobial strategies

At present, antibiotics include natural antibiotics, synthetic antibiotics, broad-spectrum antibiotics, and narrow spectrum antibiotics. They kill bacteria through different mechanisms, such as inhibiting cell walls, proteins, nucleic acid synthesis, and interfering with metabolic pathways. For fungal infections, due to the difference between fungal cell walls and bacteria, penicillins are not effective against fungi, and only a few antibiotics such as polyene antibiotics: amphotericin B [10] and nystatin. In addition, antifungal drugs mainly use azole drugs. However, with the use of antibiotics, fungal and bacterial resistance has gradually emerged.

The clinical failure of last-resort antibiotics is now driven by three, mutually reinforcing resistance mechanisms that have become ubiquitous among ESKAPE pathogens. (i) Enzymatic destruction: β-lactamases (TEM, CTX-M, KPC, NDM, VIM, IMP), aminoglycoside-modifying enzymes (aac, aph, ant) and macrolide esterases (ere) chemically inactivate the drug before it reaches its target [64]. (ii) Target-site remodeling: acquisition of mecA/mecC (MRSA) or PBP1b-L169P in penicillin-resistant Streptococcus pneumoniae lowers β-lactam affinity; 23 S rRNA methylases (erm) and point mutations (gyrA-S83F, parC-S80I) protect DNA gyrase and topoisomerase from fluoroquinolones [65,66]; substitution of D-Ala by D-Lac or D-Ser in peptidoglycan precursors produces vancomycin-resistant enterococci (VanA/B/M). (iii) Active efflux and permeability suppression: tripartite pumps (AcrAB-TolC, MexAB-OprM, AdeABC, KpnEF) expel multiple drug classes, while loss of the OmpF/K porin or addition of PagP-mediated lipid A modification shrinks the outer-membrane pore below the antibiotic's hydrodynamic radius [67]. Because every clinically deployed antibiotic ultimately relies on a single, high-affinity interaction that can be subverted by one of the above strategies, the only universally conserved counter-measure is to shift from "lock-and-key" specificity to multi-site, physicochemical insult—precisely the mode of action exploited by nanozymes.

Unlike conventional antibiotics that rely on single-target engagement and are rendered ineffective by the canonical resistance triad, nanozymes exploit catalytic, ROS-mediated, multi-site damage that circumvents all three escape routes simultaneously. Because their bactericidal action originates from surface-confined Fenton-like, POD-like or OXD-like chemistry, the lethal species (•OH, ^1^O_2_, O_2_^−^) indiscriminately oxidize membrane lipids, proteins and DNA without the "lock-and-key" recognition that bacteria can evolve to block. Recent demonstrations corroborate this conceptual advantage: V_2_O_5_ nanowires maintain a 4 μg mL^−1^ MBC against NDM-1-positive Klebsiella pneumoniae—32-fold below meropenem's minimum inhibitory concentration (MIC) for the same strain [68]—while Mn_3_O_4_, Cu_2_MoS_4_ and single-atom Fe–N–C platforms eradicate MRSA, VRE and colistin-resistant Acinetobacter baumannii biofilms without cross-resistance to vancomycin, linezolid or tigecycline. Yet the translational roadmap is still incomplete. Serial-passage studies exceeding 30 days have not revealed >4-fold MIC increases, but proteomic snapshots show transient up-regulation of oxidative-stress regulons (soxRS, oxyR), hinting at potential non-classical escape via capsule thickening or membrane charge shielding [69]; long-term evolution experiments coupled to multi-omics tracking are therefore imperative. Likewise, efficacy data are largely confined to planktonic cultures or murine models; larger animals and complex infection niches (bone, lung, diabetic wound) remain under-explored. Future efforts should prioritize (i) nanozyme–antibiotic co-formulations that exploit ROS-induced membrane permeation to lower antibiotic MICs and retard resistance selection, (ii) immuno-compatible nanozyme cloaking strategies to mitigate chronic inflammation during repeated dosing, and (iii) standardized regulatory protocols that quantify catalytic turnover number, ion leaching and ROS footprint in vivo. Addressing these gaps will determine whether nanozymes can evolve from intriguing laboratory curiosities into genuine resistance-breaking therapeutics.

### 4.2 Multidimensional sterilization mode of nanozymes and microenvironment reprogramming by nanozymes: a paradigm to bypass bacterial drug resistance

**Fig. 2:**
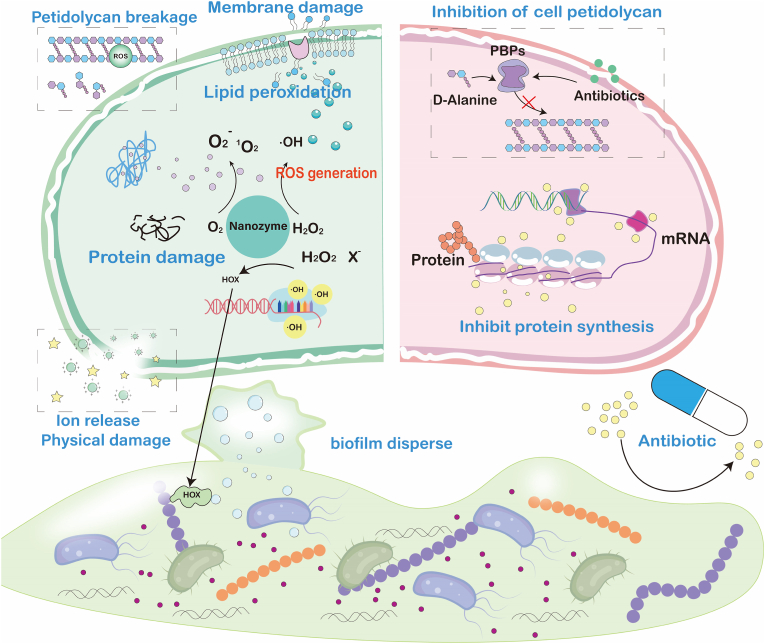
Antibacterial mechanisms: Traditional antibiotics target a single metabolic pathway, whereas nanozymes generate ROS via multi-enzyme-mimetic activities to synergistically eradicate bacteria.

As a new type of antimicrobial agent, the antimicrobial mechanism of nanozymes usually involves multiple dimensions, including simulating oxidoreductase activity, non redox mechanisms (physical damage, ion release), and microenvironmental regulation. Compared to traditional antibiotics, these unique mechanisms make it difficult for bacteria to develop resistance (Fig. 2) [70].

#### 4.2.1 ROS-mediated oxidative damage

As the core antimicrobial mechanism, nanozymes with POD and OXD-mimetic activities (Fe_3_O_4_, MnO_2_) catalyze H_2_O_2_ decomposition to generate hydroxyl radicals (•OH) via Fenton/Fenton-like reactions or singlet oxygen (^1^O_2_) through oxidase pathways. These highly oxidative ROS species: 1) Attack membrane lipids, inducing lipid peroxidation and membrane integrity loss; 2) Oxidize intracellular proteins, altering structure and function; 3) Damage DNA through strand breaks and base modifications Collectively disrupting essential bacterial physiological processes.

#### 4.2.2 Non-redox antimicrobial mechanisms

Beyond redox catalysis, nanozymes employ physical disruption and bioactive ion release as critical antimicrobial strategies. Positively charged CeO_2_ nanozymes exploit electrostatic attraction to adhere to negatively charged bacterial membranes (P aeruginosa), where progressive structural disorganization culminates in membrane lysis-achieving>95 % eradication efficacy. Concurrently, morphologically engineered nanozymes with nanosharp edges physically penetrate cellular membranes, inducing lethal cytoplasmic leakage. Metallic variants (Ag^+^, Cu^2+^ generators) further compromise bacterial viability through ion-mediated membrane disruption, protein denaturation, and metabolic interference [86].

#### 4.2.3 Targeted biofilm disruption

Biofilm-associated infections originate from highly organized microbial communities adherent to surfaces, including natural substrates, metals, polymers, biomedical implants, and human tissues. These structures are encased within an EPS matrix—comprising lipids, DNA, proteins, and polysaccharides—secreted by the microorganisms themselves. The resultant three-dimensional architecture confers intrinsic protection to embedded pathogens.

The biofilm microenvironment exhibits marked spatiotemporal heterogeneity, characterized by gradient-driven variations: pH partitioning (acidic microenvironments) Oxygen-depleted niches, Nutrient-limiting zones, Elevated expression of antioxidants (e.g., GSH) Concurrently, biofilm-resident bacteria utilize QS to synchronize gene expression, forming stratified, antibiotic-tolerant consortia. These collective attributes underpin the failure of conventional antimicrobial therapies.

Overcoming limitations of conventional antibiotics, nanozymes leverage their nanoscale dimensions to penetrate biofilm matrices via diffusion. Beyond physical infiltration, they specifically target critical biofilm components: (1) Degrading extracellular DNA (eDNA) through deoxyribonuclease (DNase)-mimetic activity, destabilizing structural integrity. Ultra-small cerium-based metal-organic framework (Ce-MOF) nanozymes exhibit dual enzyme-like activities: DNase-mimetic Ce(IV) sites hydrolyze eDNA to disrupt mature biofilm architecture; POD-like activity catalyzes H_2_O_2_-derived ROS generation, oxidizing protein thiols, cleaving polysaccharide chains, and damaging eDNA to suppress biofilm regeneration [87]. Given the complexity of biofilm infections, single-mechanism approaches often prove inadequate. Integrated theranostic strategies are exemplified by Lei Han's team: Aggregation-induced emission Artifical Enzyme (AIEzymes) (Zr-based coordination polymer nanoparticles) enable: Sustained eDNA hydrolysis for deep biofilm eradication; Real-time fluorescence monitoring of material distribution and metabolism at infection sites via rigid fluorophore properties [88]. (2) Inhibiting QS by catalyzing HOBr formation from H_2_O_2_ and halides [[89], [90], [91]]. Inspired by marine antifouling mechanisms, CeO_2_−_x_ nanorods mimic vanadium haloperoxidases (V-HPOs) to catalyze H_2_O_2_ and Br^−^ transformation into hypobromous acid (HOBr). This selectively modifies quorum-sensing molecules (e.g., N-acyl homoserine lactones, AHLs), disrupting signal transduction and inhibiting biofilm formation at its inception [92]. Such biomimetic catalytic approaches establish novel paradigms for non-biocidal *anti*-biofilm therapeutics.

#### 4.2.4 Microenvironment regulation

Bacterial infection microenvironments exhibit distinct pathological features—including acidic pH, hypoxia, hyperglycaemia, and immunosuppression—that diverge from physiological conditions. Nanozymes combat infections not only through direct bactericidal actions but also via microenvironment modulation. For diabetic wound infections, GOx-nanozyme cascades simultaneously: (1) Alleviate hyperglycaemia through glucose consumption. (2) Acidify infected sites via gluconic acid generation. (3) Produce endogenous H_2_O_2_ as antimicrobial agent.

Hypoxic biofilm interiors are addressed through oxygen-independent oxidase-mimetic nanozymes that generate ROS without O_2_ dependency, overcoming PDT limitations. Complementary strategies include GPx-mimetic nanozymes that deplete bacterial GSH reserves, compromising oxidative stress defenses and enhancing ROS lethality. Single-atom bionanozymes (BioSAzyme) employ a triple-functional design: 1) Biorecognition: Lectin (ConA) mediates specific binding to biofilm glycocalyx for spatial targeting; 2) Metabolic Activation: Integrated GOx depletes endogenous glucose, concomitantly generating H_2_O_2_ and gluconic acid to achieve self-sufficient ROS precursors and microenvironment acidification; 3) Cascade Catalysis: Engineered catalytic sites amplify ROS production [93].

#### 4.2.5 The synergistic and Cascade Catalysis of nanozymes

The pronounced advantage of nanozymes lies in their multifunctional characteristics. A single nanozyme can concurrently harbor multiple enzymatic activities as well as other non-enzymatic antimicrobial mechanisms (including the previously mentioned non-redox mechanism). This multifunctionality empowers nanozymes to exert potent antimicrobial effects through synergistic actions of different functions: (1) ROS generation, synergistic physical disruption, and ion release. (2) ROS generation working in synergy with hydrolytic enzyme activity to degrade biofilms. (3) Self-cascading of multi-enzyme activities.

Moreover, nanozymes can be integrated with other therapeutic approaches. For instance, ROS generation can be enhanced in combination with photothermal therapy (PTT) and sonodynamic therapy (SDT) [94]. Additionally, nanozymes can synergize with other enzymes (such as GOx) to form a cascade reaction. Take the example of GOx catalyzing glucose to produce H_2_O_2_, which is subsequently converted into •OH by POD-like activity, thereby achieving a dual effect of "sugar consumption and sterilization" [95].

The antimicrobial activity of nanozymes is a highly integrated and dynamic process. Its core lies in utilizing its enzyme like catalytic activity (especially ROS production) as the main weapon, improving delivery efficiency through design modifications, and providing diversified attack methods by combining non redox mechanisms (physical destruction, ion release, enzymatic hydrolysis). The most crucial thing is that nanozymes can trigger multiple mechanisms simultaneously or sequentially, producing powerful synergistic effects. In addition, the design can enable nanozymes to intelligently respond to unique microenvironments (acidic, hypoxic, high H_2_O_2_/GSH) to enhance their own activity and specificity. This multidimensional, multi mechanism, and responsive antimicrobial strategy makes nanozymes a highly promising new generation of antimicrobial agents for combating the increasingly serious problems of bacterial resistance and biofilm infections.

## 5 Application of nanozymes in disease models

In the field of anti infection, nanozymes have shown great potential. However, different infection models have unique pathogen characteristics and microenvironments of infection sites, which require targeted adjustments to the composition, surface modification, and mechanism of action of nanozymes to achieve optimal antibacterial effects.

### 5.1 Local infection models

**Fig. 3:**
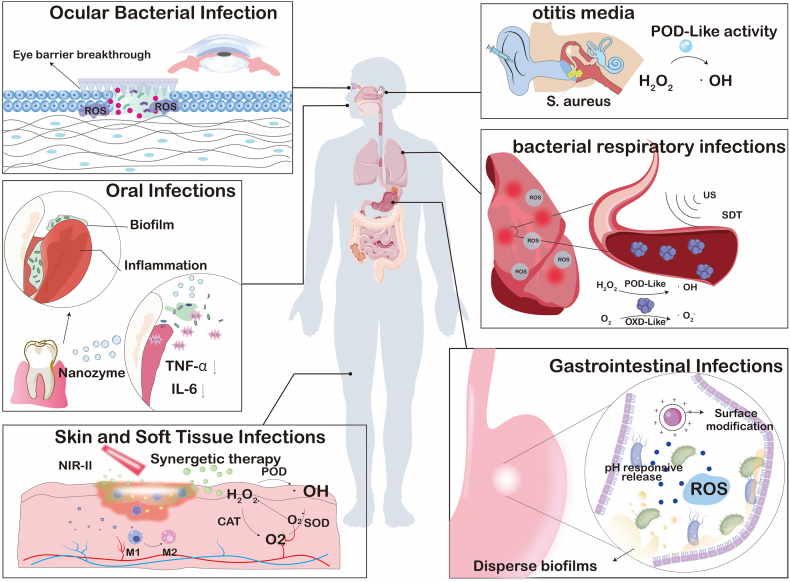
Schematic of nanozyme therapy for localized bacterial infection.

Nanozymes, with their various enzyme activities, have reshaped the "antibiotic free" antibacterial paradigm in the treatment of local infections. Its core idea is to integrate inorganic or metal organic nano enzymes with peroxide, oxidation or halogen activities into hydrogels, microneedles and other applicable or injectable carriers. By taking advantage of the characteristics of low pH, high H_2_O_2_ and high GSH in the infected microenvironment, it can catalyze the production of living oxygen in situ, causing multiple damage to bacterial membranes, proteins and nucleic acids, while destroying biological membranes and blocking drug resistance pathways. These drug delivery systems precisely release enzymes at the lesion site through metal coordination, electrostatic or enzyme response, maintaining high local concentrations and significantly reducing systemic exposure; some designs also couple glucose oxidation, photothermal or photodynamic units to achieve multi-mechanism synergy, further enhancing the depth and speed of sterilization (Fig. 3).

#### 5.1.1 Skin and soft tissue infections

**Fig. 4:**
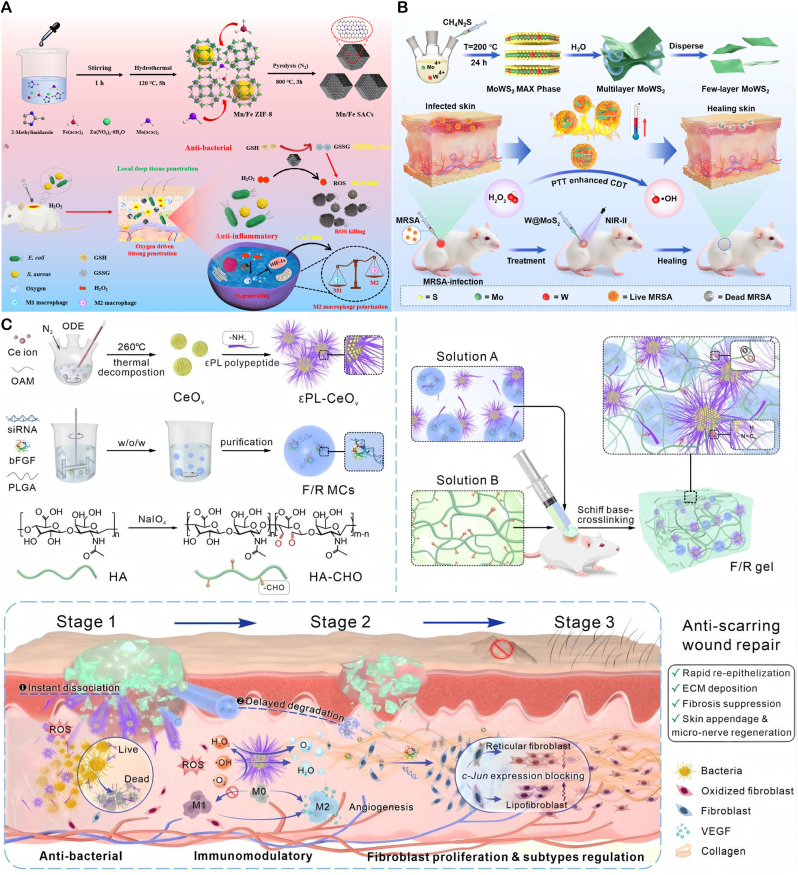
(**A**) Schematic of Mn/Fe–ZIF-8 synthesis to Mn/Fe SACs and their CAT/POD/GSHOx-like therapy for bacterial wound infection. Copyright 2023 American Chemical Society. (**B**) Schematic of few-layer MoWS_2_ preparation and 1064 nm-laser-enhanced POD-like activity promoting in vivo healing of *S. aureus* abscesses. Copyright 2024 Acta Materialia Inc. (**C**) Schematic diagram of F/R gel promoting cell proliferation and angiogenesis by releasing VEGE. Copyright 2025 Springer Nature Limited.

Skin and soft tissue infections, often caused by pathogens like *S. aureus* and Streptococcus spp., are common. These bacteria's strong adhesion and biofilm-forming capabilities make infections difficult to eradicate. Moreover, the skin's barrier function and relatively dry environment pose challenges for nanozyme penetration and action. To address these infections, nanozyme design must focus on efficient bactericidal activity, promoting wound healing, and ensuring good biocompatibility. A systematic integration of multi - dimensional functions is essential, and can be explored through the following key dimensions:

**Multi-enzyme Synergistic Bactericidal Activity** Design composite nanozyme systems, such as Fe_3_O_4_@MnO_2_ core-shell structures, to mimic POD-like, CAT-like, and SOD-like activities. In the slightly acidic (pH 5.5–6.5) and high-H_2_O_2_ (10–100 μM) environment of infected sites, CAT-like activity breaks down H_2_O_2_ to produce O_2_, alleviating local hypoxia. POD-like activity catalyzes H_2_O_2_ to generate highly toxic •OH (bactericidal rate >95 %), while SOD-like activity converts superoxide anions to H_2_O_2_, forming a cycle to prolong the bactericidal effect. Zhang's team developed Mn/Fe single-atom catalysts (Mn/Fe SACs) with multi-enzyme activity, which can: 1) catalyze H_2_O_2_ to generate ROS, inducing CDT to eliminate bacteria at infected sites; 2) decompose H_2_O_2_ to produce oxygen and enhance tissue penetration; and 3) regulate macrophage polarization to the anti-inflammatory M2 phenotype. In a mouse model of *S. aureus* infection, Mn/Fe SACs significantly accelerated wound healing and suppressed inflammatory responses through immunoregulation(Fig. 4A) [96].

**Combination Therapy for Enhanced Bactericidal Effects** A tungsten-doped defective 2H-MoS_2_ nanozyme (MoWS_2_), prepared by hydrothermal method, was reported to promote wound healing in MRSA-infected sites (Fig. 4B) [97]. MoWS_2_ combines PTT with enzyme-catalytic action, enhancing catalytic activity through local hyperthermia. Tungsten doping significantly improved the material's near-infrared second window (NIR-II) absorption and enzyme-catalytic performance. In vitro, MoWS_2_ efficiently killed MRSA and removed biofilms through high temperatures and ROS. In a deep MRSA infection model, MoWS_2_ rapidly cleared subcutaneous abscess bacteria and accelerated tissue healing through the synergistic effects of PTT and CDT. Multi-mechanistic treatment modes of MoWS_2_ were further confirmed by tissue sectioning and immunofluorescence staining analyses.

**Microenvironment Regulation for Wound Repair** nanozymes can be designed to dynamically regulate the "anti-inflammatory-pro-repair" process during infection-associated inflammation and tissue regeneration Inflammatory Phase Regulation: Graft IL-10 antibodies or TGF-β inhibitory peptides to specifically neutralize over-secreted pro-inflammatory factors (TNF-α, IL-6) in the early infection phase (1–3 days). This polarizes M1 macrophages to the M2 type, reducing the risk of local cytokine storms. Simultaneously, GPx-like activity clears excessive ROS to prevent oxidative damage to normal tissues. For example, a platinum nanozyme-engineered probiotic microneedle patch has been demonstrated in diabetic wound models to effectively reduce levels of pro-inflammatory factors IL-6 and TNF-α, and promote the polarization of macrophages from pro-inflammatory M1 to anti-inflammatory and reparative M2 phenotypes. It exhibits SOD-like and CAT-like activities, efficiently clearing accumulated ROS in the wound, protecting cells from oxidative damage, and creating favorable conditions for subsequent repair. Another study on a trimetallic nanozyme-based smart hydrogel also showed that it can significantly downregulate M1 markers (IL-6, TNF-α) while upregulating M2 markers (IL-10, TGF-β1), thereby improving the repair microenvironment [98]. Proliferation Phase Promotion: Studies have found that in the treatment of diabetic foot ulcers, elevated levels of bFGF (basic fibroblast growth factorand VEGF (vascular endothelial growth factor) are significantly associated with granulation tissue growth and accelerated wound healing. Earlier animal experiments have also demonstrated that local application of VEGF not only promotes angiogenesis but also improves microcirculation and enhances the anti-infection capacity of skin flaps. Sustained-release carriers loaded with bFGF or VEGF can continuously release growth factors after inflammation subsides (3–7 days), promoting fibroblast proliferation and collagen deposition. The team led by Li Xiaokun at Wenzhou Medical University prepared an F/R hydrogel by mixing cationic polypeptide (ε-polylysine, εPL) and aldehyde-modified hyaluronic acid (HA-CHO) via Schiff base cross-linking reaction. The hydrogel was embedded with εPL-modified oxygen-deficient nanoceria (εPL-CeOv) and PLGA microcapsules loaded with basic fibroblast growth factor (bFGF) and c-Jun siRNA. The F/R hydrogel can rapidly degrade in acidic environments (such as bacterial biofilm environments), releasing low-molecular-weight εPL and ultrasmall εPL-CeOv, which exert antibacterial and antioxidant effects. As the hydrogel degrades, the F/R microcapsules gradually release bFGF and c-Jun siRNA, promoting cell proliferation and angiogenesis (Fig. 4C) [99].

#### 5.1.2 Oral infections

Oral infections, triggered by diverse bacteria like Streptococcus mutans, Porphyromonas gingivalis, and Aggregatibacter actinomycetemcomitans [126], are difficult to eliminate due to the oral cavity's unique physiology. This environment facilitates bacterial colonization and biofilm formation within the intricate root canal system, leading to complications such as caries, chronic gingivitis, and periodontitis. Traditional antibiotics often fall short in effective treatment because of saliva-induced drug dilution, poor drug penetration into deep-seated infections, short-duration drug action, and biofilm formation. In contrast, nanozymes, with their distinct antibacterial mechanisms, tunability, and adaptability, show great potential for effectively treating oral infections. Researchers have developed various nanozymes to address the challenges posed by oral infections.

**Fig. 5:**
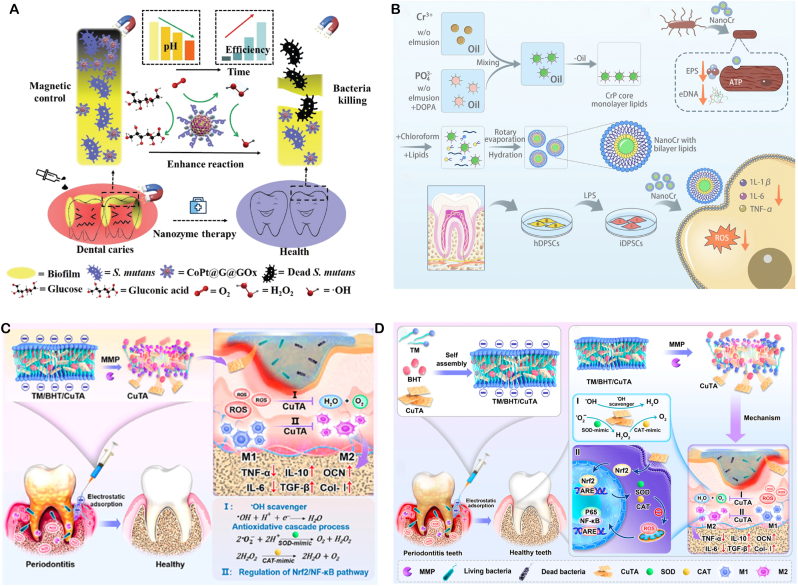
(**A**) Proposed mechanism of a hybrid nanozyme that disrupts oral pathogenic biofilms via an enzyme–nanozyme cascade, delivering both antibacterial and antibiofilm actions. Copyright 2025 Springer Nature. (**B**) Schematic overview of NanoCr synthesis, its antibacterial activity, and anti-inflammatory modulation of inflammatory DPSCs. Copyright The Royal Society of Chemistry 2023. (**C,D**) Schematic illustration of the TM/BHT/CuTA hydrogel: self-assembled from CuTA nanozyme, TM and BHT for periodontitis therapy. Negatively charged TM/BHT/CuTA adheres electrostatically to positively charged inflamed tissue, enabling prolonged retention. Upon exposure to elevated MMP and ROS within plaque-induced periodontitis, the hydrogel on-demand releases CuTA. The liberated nanozyme (i) eliminates periodontitis-associated bacteria, (ii) scavenges ROS via SOD–CAT cascade, and (iii) triggers Nrf2/NF-κB signaling to shift M1 toward M2 macrophages, thereby dampening pro-inflammatory cytokines, boosting anti-inflammatory mediators and osteogenic genes, and accelerating periodontal regeneration. Copyright 2023 American Chemical Society.

**Bactericidal and Anti-inflammatory Dual - functional nanozymes** Excessive ROS production can kill bacteria but may also cause severe inflammatory reactions affecting normal tissues. Balancing ROS generation and avoiding over-oxidation is crucial in antibacterial nanozyme design. Yuanyuan Wang's team developed a core-shellstructured chromium nanozyme (NanoCr) with antibacterial, ROS-scavenging, and anti-inflammatory properties. It significantly inhibits *E. coli*, *Enterococcus faecalis*, and Porphyromonas gingivalis and mitigates ROS - induced inflammation via multi - enzyme activities. In vitro, NanoCr disrupts bacterial cell structures and metabolic pathways, reduces inflammatory factors in pulpitis models, and curbs excessive immune responses(Fig. 5B) [100]. This dual-functionality offers a new approach for pulpitis treatment.

**Stable and Controlled Release Intelligent Responsive nanozymes** Smart responsive nanozymes can address poor patient compliance and insufficient drug concentrations. Wang's team developed a copper-based nanozyme hydrogel system, copper tannic acid nanosheets (CuTA NSs), which can target and inhibit oral pathogens like Fusobacterium nucleatum and *S. aureus*. They further constructed an MMP-responsive TM/BHT/CuTA hydrogel system that smartly releases CuTA nanozymes based on matrix metalloproteinase (MMP) levels in the periodontitis microenvironment. This hydrogel system has shown good antibacterial activity, osteogenic performance, and biocompatibility in vitro and in vivo, providing an innovative solution to the problems of drug concentration fluctuations and poor patient compliance in traditional treatments(Fig. 5C and D) [101].

**Cascading Catalytic nanozymes** The CoPt@G@GOx designed by Dong's team enables precise antibacterial action via magnet-driven cascading reactions. Firstly, GOx catalyzes glucose to produce H_2_O_2_. Then, cobalt-platinum alloy nanozymes convert H_2_O_2_ into highly toxic •OH via a Fenton-like reaction. This material shows specific killing effect on Streptococcus mutans biofilms and can target biofilms through magnet-driven cascading reactions, reducing damage to normal tissues(Fig. 5A) [102]. This controllable catalytic system offers a new approach for treating biofilm-related oral diseases.

The aforementioned studies highlight the breakthrough potential of nanozymes in treating oral infections through multiple mechanisms, including catalytic antibacterial actions, immune regulation, and smart drug delivery. These findings provide theoretical and technical support for nanozymes as promising alternatives to traditional antibiotics.

#### 5.1.3 Ocular bacterial infection models

Bacterial infections of the eye, often caused by pathogens such as P aeruginosa, Streptococcus pneumoniae, and *S. aureus*, commonly lead to keratitis and conjunctivitis. These infections pose significant therapeutic challenges. Novel nanozyme-based antibacterial strategies have shown remarkable potential by mimicking natural enzyme activities, offering non-antibiotic treatment options. This approach not only avoids the risk of antibiotic resistance but also significantly enhances therapeutic efficacy, achieving notable progress particularly in the treatment of bacterial keratitis.

**Fig. 6:**
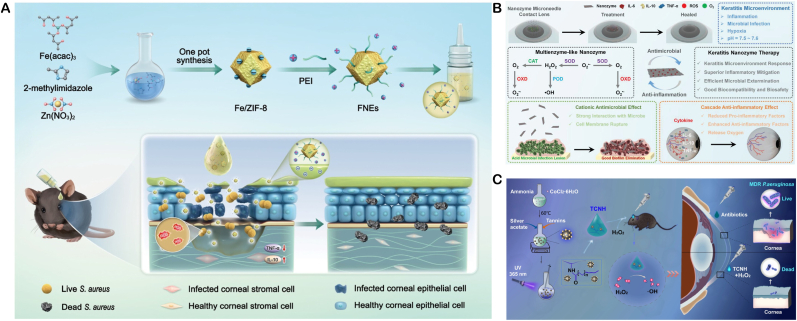
(**A**) Schematic of FNEs fabrication and their therapeutic deployment against bacterial keratitis. Copyright 2024 Wiley‐VCH GmbH. (**B**) Schematic of multi-enzyme-mimetic nanozyme-loaded ocular microneedles for infectious-keratitis therapy. Copyright 2023 Wiley-VCH GmbH. (**C**) Schematic of tannin-coordinated nanozyme-hybrid hydrogel eye drops for preventing MDR *P. aeruginosa* keratitis. Copyright 2025 BioMed Central Ltd unless otherwise stated. Part of Springer Nature.

In terms of material development, the iron doped nanozyme system (Fe^3+^-doped nanozymes, FNEs) constructed by the Geng team is innovative. This system is based on Fe^3+^ doped Fe/ZIF-8 as the core, combined with polyethyleneimine (PEI) to form a stable composite, which also has excellent water dispersibility and biocompatibility. Its mechanism of action originates from spontaneous POD activity, which can catalyze H_2_O_2_ to generate high concentrations of ROS in the infected microenvironment, effectively killing pathogens through oxidative stress effects. Animal experiments have shown that the nanozyme achieves complete clearance of *S. aureus* in a mouse bacterial keratitis model within 6 h. Its anti-inflammatory effect and pathogen clearance efficiency are significantly better than traditional antibiotic eye drops, and no drug resistance was observed (Fig. 6A) [103].

The multifunctional microneedle system (MnOx) developed by Liu's team aims to break through the barrier of ocular drug delivery (GDY@MGMN) has technological breakthrough significance. This system combines corneal penetration and sustained release properties by encapsulating manganese oxide/graphite diyne composite nanozymes (MnOx/GDY) in hyaluronic acid polymethyl methacrylate based microneedles (MGMN). In vitro and in vivo experiments have confirmed that the microneedle system can not only effectively eliminate pathogens and inhibit biofilm formation, but also promote corneal repair by improving the hypoxic microenvironment of tissues. Its therapeutic effect has significant advantages over the antifungal drug voriconazole and demonstrates good biocompatibility (Fig. 6B) [104].

For the prevention and treatment needs of multi drug resistant bacteria, the tannin coordination nano enzyme composite hydrogel (TCNH) developed by Hongwei Wang's team provides innovative solutions. This preparation is a hybrid system constructed through molecular coordination engineering, which combines POD-like activity with tissue adaptability. The experiment proved that it has a significant inhibitory effect on the standard strain of P aeruginosa and clinical multi drug resistant strains, and at the same time, it can realize the persistent residence of drugs on the eye surface through the hydrogel carrier. This study has opened up new avenues for the treatment of drug-resistant eye infections(Fig. 6C) [105].

Current research highlights the substantial clinical potential of nanozyme technology for treating ocular infections. Compared with traditional antibiotics, its key advantages are: (1) evasion of drug resistance through ROS-mediated, non-specific bacterial killing; (2) improved drug bioavailability conferred by nanocarrier systems; and (3) multifunctional integration—combining antibacterial, anti-inflammatory, and tissue-repair activities—for synergistic therapy. These attributes are essential for the development of next-generation, smart ocular anti-infection formulations.

#### 5.1.4 Gastrointestinal infections

*H. pylori*, an acid - tolerant microaerophile, adheres to the gastric mucosa and induces chronic inflammation. With a global infection rate of 50 % and up to 59 % in China, it is closely linked to dyspepsia, gastric ulcers, and a 4-to 6-fold increased risk of gastric cancer. Current quadruple therapy (two antibiotics plus a proton pump inhibitor) has drawbacks like lengthy treatment duration, disruption of gut microbiota, and rising drug resistance. Nanozymes, with their excellent pH adaptability, can remain active in gastric acid and digestive enzymes. Their tunable and designable properties offer new possibilities for overcoming the limitations of treating such bacterial infections.

**Fig. 7:**
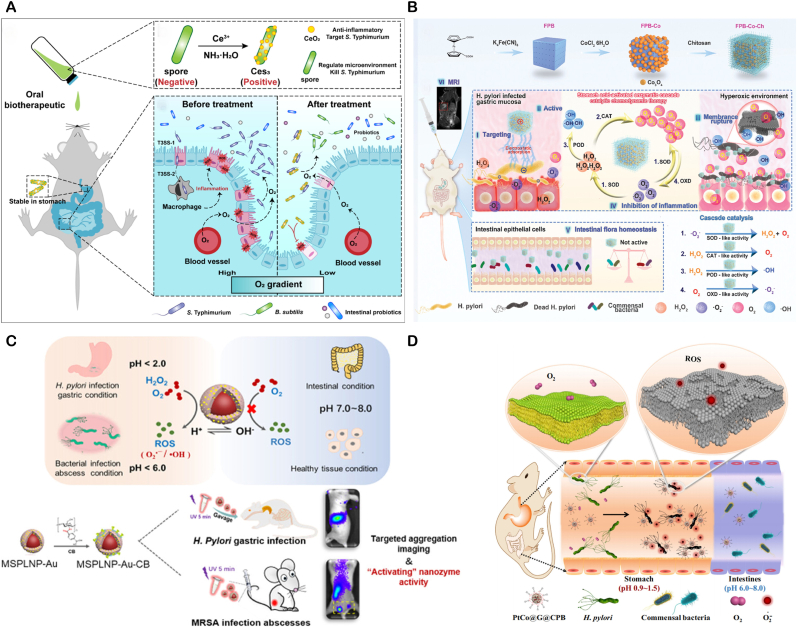
(**A**) Schematic of FPB-Co-Ch nanozyme fabrication and its gastric-to-intestinal journey: (i) chitosan-mediated *H. pylori* targeting, (ii) cascade OXD, SOD, POD, CAT activation for bactericidal action, (iii) membrane disruption and selective killing, (iv) inflammation alleviation, (v) maintenance of intestinal flora homeostasis, and (vi) in vivo MRI capability. Copyright 2024 Wiley-VCH GmbH. (**B**) Ces3 oral biotherapeutic—formed by in-situ CeO_2_ nanozyme growth on spores—targets S. Typhimurium, scavenges ROS, and depletes O_2_ in the ROS-rich, O_2_-spiked microenvironment of acute gastroenteritis, thereby eradicating pathogens, repairing mucosal damage, and restoring intestinal microbiome balance. Copyright 2024 American Chemical Society. (**C**) Schematic of MSPLNP-Au-CB: selectively killing and imaging bacteria in gastric/micro-acidic milieu while remaining innocuous to normal cells and commensal flora under physiological conditions. Copyright 2021 American Chemical Society. (**D**) Schematic of PtCo@G@CPB nanozyme–mediated in vivo selective sterilization: in acidic stomach, precise *H. pylori* targeting and ROS catalysis eradicate the pathogen; upon transition to neutral intestine, oxidase-like activity is quenched, sparing commensal microbes. Copyright 2025 Springer Nature Limited.

**Targeted nanozymes for Reduced Tissue Damage** Nanozymes may harm normal tissues. To address this, researchers employ surface modification with targeting - capable coatings. For example, Rimo Xi's team developed a multi-enzyme nano-system targeting *H. pylori*, the cobalt - based Prussian blue nanozyme modified with chitosan (FPB-Co-Ch). In the gastric acid environment, it activates OXD-like, SOD-like, POD-like, and CAT-like activities. Through cascading reactions, it generates O_2_ and H_2_O_2_ for efficient bactericidal action. Its activity is suppressed in the intestine's neutral environment, reducing toxicity to commensal bacteria and minimizing damage to normal physiological tissues and probiotics (Fig. 7A) [106].

**Probiotic delivery combined with enzyme catalyzed therapy** The gut is home to a diverse community of bacteria. During bacterial infections, beneficial bacteria often struggle to compete and may be suppressed. When treating gastrointestinal infections, it's crucial to eliminate pathogens while protecting these beneficial bacteria. Nanozymes, with their remarkable enzymatic activity, can serve as antibacterial agents. Additionally, as nanomaterials, they can be used for drug delivery. This has inspired researchers to use nanozymes to deliver probiotics, introducing new beneficial bacteria while eliminating harmful ones. For instance, Wei's team developed a probiotic-coordinated CeO_2_ nanozyme (Ces3). Ces3 is grown in situ on probiotic spores and can bind to gut pathogens like Salmonella via electrostatic interactions. It protects probiotics from oxidative stress by scavenging ROS and promotes the growth of anaerobic probiotics by reducing oxygen levels, thereby restoring gut microbiota balance. In a mouse model of acute gastroenteritis, Ces3 effectively inhibited Salmonella infection while maintaining gut micro-ecological homeostasis (Fig. 7B) [107].

***In vivo* imaging combined with enzyme catalyzed therapy** Controlling the activation of nanozymes in specific areas is crucial for enhancing their precision. Given the significant pH variations in the gastrointestinal tract, designing a pH-responsive luminescent nanozyme platform ensures nanozymes act under specific pH conditions, focusing their activity and improving stability in complex environments. The MSPLNP-Au-CB nanozyme, composed of porous silicon-based luminescent particles, gold nanoparticles, and a chitosan-phenylboronic acid complex, features: 1) Persistent luminescent imaging. 2) Acid-specific release from phenylboronic acid modification. 3) Bacterial-targeting adhesion. It generates ROS specifically in gastric acid and the slightly acidic environment of MRSA infection, without harming normal tissues. In a mouse model of *H. pylori* infection, this system achieved precise bacterial elimination and real-time imaging (Fig. 7C) [108].Another example is the PtCo@graphene nanozyme (PtCo@G@CPB), which has enhanced targeting of *H. pylori* with the help of CPB molecules on its surface. In gastric acid, it activates OXD-like activity to produce ROS and kill bacteria. However, it has no antibacterial effect under the neutral conditions of the intestine. In vitro, PtCo@G@CPB shows high antibacterial activity against *H. pylori* with minimal side effects on normal tissues and commensal bacteria. In a mouse model of *H. pylori* infection, it exhibits efficient bactericidal ability with negligible impact on normal tissues and commensal bacteria. Moreover, it has magnetic resonance imaging and Raman imaging capabilities, enabling real-time monitoring of the nanomaterial's distribution in vivo. When activated in vivo, PtCo@G@CPB selectively clears *H. pylori* without affecting commensal bacteria (Fig. 7D) [109].

**POD-Mediated Autophagy for Salmonella Elimination** Shi's team demonstrated that iron oxide nanozymes (IONzymes) mediate autophagy pathways via POD-like activity to inhibit Salmonella Enteritidis. In vitro experiments showed that IONzymes colocalize with pathogens within autophagosomes in host cells, increasing bacterial clearance through elevated ROS levels. In vivo, orally administered IONzymes reduced bacterial loads in the livers of specific-pathogen-free (SPF) chickens and prevented tissue damage, offering a new strategy for controlling gastrointestinal infections in poultry [110].

Nanozymes, as alternatives to traditional antibiotics, have shown great antibacterial potential. Researchers have explored their enzyme activity regulation, microenvironment response, and microbiota protection. These efforts have overcome traditional antibiotic therapy limitations and opened new avenues for precise gastrointestinal infection treatment.

#### 5.1.5 Nanocatalytic therapy for bacterial respiratory infections

Bacterial respiratory tract infections primarily affect the lower respiratory system below the larynx, clinically manifesting as pneumonia, bronchitis, and infections complicating bronchiectasis. Common pathogens include *S. aureus*, P aeruginosa, and Streptococcus pneumoniae. These bacteria often form biofilms within the respiratory tract, impeding the efficacy of antibiotics. Moreover, the emergence of multidrug-resistant strains, such as MRSA, has significantly diminished the effectiveness of traditional antibiotic therapies. In recent years, nano-catalytic materials have emerged as a unique and promising strategy for treating these infections.

To tackle infections caused by MDR bacteria, Hong Yao's team developed a bimetallic BiPt nanozyme system. They used platelet - bacterial hybrid membranes (BiPt@HMVs) to enhance the nanozymes' targeting and bacterial adhesion. Ultrasound irradiation boosts its catalytic activity, promoting efficient ROS generation. This system effectively targets MDR pathogens like CRE and MRSA, showing significant therapeutic effects in pneumonia, osteomyelitis, and muscle infection models. This ultrasound - enhanced nanocatalytic therapy offers an innovative solution for clinical MDR bacterial infections, and its targeted delivery design is highly valuable for nanodrug development (Fig. 9A) [127].

Bacterial respiratory infections often trigger severe inflammatory responses, which can cause significant damage to the respiratory system. Antibiotic therapy is limited in controlling inflammation. Nanozymes, with their multiple enzymatic activities, can exert both antibacterial and anti-inflammatory effects, maintaining redox balance and modulating the immune microenvironment.

Zinc-based hexacyanoferrate (II) nanocatalysts (ZnPBA NCs) have shown a synergistic antibacterial-anti-inflammatory mechanism in treating bacterial pneumonia. Studies indicate that ZnPBA NCs possess concentration-dependent antibacterial activity against common pneumonia-causing bacteria such as *E. coli*, *S. aureus*, *Klebsiella pneumoniae*, and P aeruginosa. At a concentration of 100 μg/mL, they significantly reduce bacterial survival, demonstrating broad-spectrum antibacterial properties. They work by disrupting bacterial cell membrane structures and inducing cytoplasmic efflux, leading to bacterial death. Moreover, ZnPBA NCs effectively regulate acute inflammatory responses caused by bacterial pneumonia. Experimental data shows they can markedly lower pulmonary inflammatory cytokine levels and reduce pathological damage. This dual - functional mechanism of ZnPBA NCs provides a theoretical basis for developing new pneumonia treatment strategies and shows potential for clinical translation [128].

#### 5.1.6 Nanocatalytic therapy for otitis media

Otitis media, often due to eustachian tube dysfunction during upper respiratory viral infections, has a 50 %–90 % bacterial isolation rate in middle ear fluid, with common bacteria including Streptococcus pneumoniae, Haemophilus influenzae, and Moraxella catarrhalis. Antibiotic treatment faces challenges such as bacterial drug resistance and insufficient drug concentrations due to poor penetration of the tympanic membrane. Nanozymes, with their small size, can penetrate the tympanic membrane. Their nonspecific antibacterial mechanism effectively targets drug - resistant bacteria, addressing these treatment challenges.

To address *S. aureus* - induced otitis media (OM), Wang's team developed a ruthenium-based single-atom nanozyme (Ru-C_3_N_4_). This material incorporates four antibacterial metals—silver, iron, copper, and ruthenium—loaded onto a crystalline graphitic carbon nitride (g-C_3_N_4_) substrate. Among these, Ru-C_3_N_4_ exhibits the highest POD-like activity, with a catalytic efficiency comparable to that of natural HRP. In vitro antibacterial assays reveal that Ru-C_3_N_4_ markedly inhibits *S. aureus* proliferation at extremely low concentrations (0.06 mg/mL) and shows narrow-spectrum antibacterial activity specific to Gram-positive bacteria. Additionally, its high catalytic activity enables it to effectively degrade biofilms formed by *S. aureus*, surmounting bacterial drug-resistance barriers. In an OM mouse model, Ru-C_3_N_4_ not only eradicates pathogens but also alleviates ear inflammation. In contrast, untreated mice progress to irreversible hearing loss. Notably, the low metal loading design ensures no significant toxicity in blood, liver, or kidneys, underscoring its favorable biosafety profile (Fig. 9C) [129].

#### 5.1.7 Nanocatalytic intervention for urinary system infections

UTIs are categorized into upper urinary tract infections (e.g., pyelonephritis) and lower urinary tract infections (e.g., cystitis, urethritis). Ascending infection is the primary route, with *E. coli* accounting for over 80 % of cases. Standard UTI treatment typically involves fluoroquinolones or trimethoprim - sulfamethoxazole, yet these approaches are often associated with low cure rates and extended treatment durations. Recent research has seen significant breakthroughs in using nanozymes for UTI treatment. Researchers have developed various nanozyme systems tailored to different infection scenarios.

**Antibacterial Coating Design:** CAUTI, a prevalent hospital - acquired infection caused by biofilm - forming bacteria on catheter surfaces, poses significant clinical challenges due to the bacteria's enhanced antibiotic tolerance. To address this, Limin Shang's team developed an Au/Fe-Ag_2_O_2_ NPs - enhanced hydrogel coating. This coating, applied to silicone catheters, exhibits both POD-like and CAT-like activities. It simultaneously delivers antibacterial, *anti*-biofilm, and anti-inflammatory effects within the urinary microenvironment. The hydrogel improves catheter hydrophilicity, enhancing patient comfort. In vitro and in vivo experiments confirmed its multifaceted functions: inhibiting bacterial colonization, preventing biofilm formation, and modulating immune responses. This innovative strategy highlights the potential of multi-catalytic nanozymes in managing complex infection microenvironments and offers a novel solution for CAUTI prevention and treatment (Fig. 9B) [26].

**Enzyme-Catalytic and Biofunctional Biomimetic Systems:** Zhang's team designed a dextran - coated cerium nanobiozyme (DEC) system. DEC mimics the function of urinary protein UMOD to block bacterial FimH protein from binding to host cells. Simultaneously, the cerium nanoparticles' antioxidant properties inhibit inflammatory cascades. In mouse models of acute UTI, recurrent UTI, and CAUTI, this "nano-trap" reduced bacterial colonization and tissue damage. Effective against multidrug-resistant UTI pathogens, DEC also shows potential for FimH-mediated infections in other organs like the lungs and gut. This marks significant progress in nanozyme technology for multi-organ infection treatment [130].

### 5.2 Nanocatalytic intervention for systemic infections

**Fig. 8:**
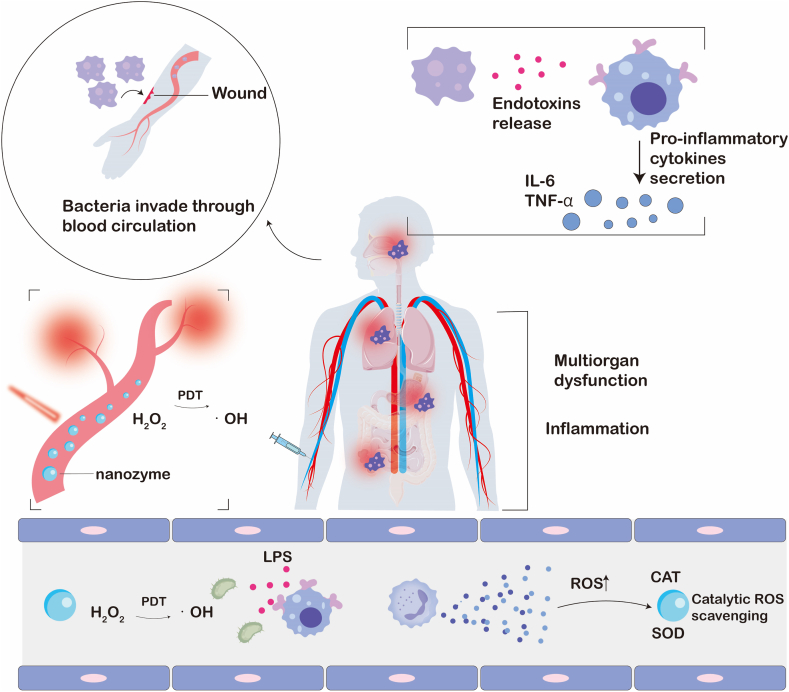
Schematic diagram of systemic inflammatory response caused by bacterial infection and nanozyme mediated treatment and anti-inflammatory of bacterial infection.

Systemic infection refers to a pathological condition where pathogens disseminate throughout multiple organs via the bloodstream. Unlike localized infections with single-focus pathology, systemic infection can lead to multisystemic damage and life-threatening complications. When pathogens, such as bacteria or viruses, breach local defenses and enter the bloodstream, they trigger a systemic inflammatory response. Clinically, this may manifest as fever, exaggerated inflammatory responses, systemic fatigue, and organ dysfunction. If not timely intervened, this condition may deteriorate into critical states like organ failure and septic shock(Fig. 8).

**Fig. 9:**
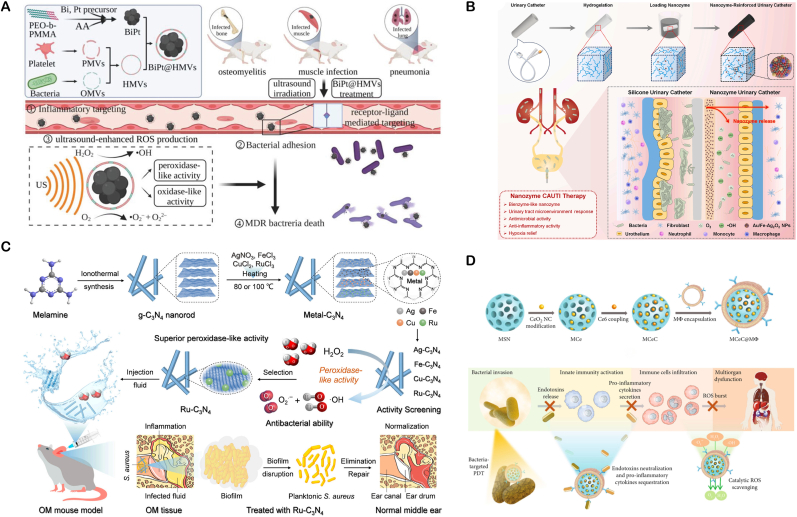
(**A**) Schematic of BiPt@HMVs: platelet–bacteria hybrid-membrane-coated BiPt nanozymes home to infection sites via platelet tropism (1) and bind pathogens through bacterial-membrane recognition (2); ultrasound amplifies BiPt POD/OXD activity, triggering lethal ROS bursts (3) against CRE- and MASR-induced infections in osteomyelitis, muscle, and pneumonia models. Copyright 1999–2025 John Wiley & Sons, Inc or related companies. (**B**) Schematic of nanozyme-reinforced urinary catheter: fabrication and on-site catalytic eradication of CAUTI. Copyright 2024 Elsevier Ltd. (**C**) Schematic of metal-C_3_N_4_ synthesis and its therapeutic application in a S. aureus–induced osteomyelitis model. Copyright 2025 Frontiers Media S.A. (**D**) Schematic of MCeC@MΦ decoy nanozymes: fabrication and multitarget suppression of pro-inflammatory cascades in MDR bacterial sepsis. Copyright 2025 American Association for the Advancement of Science.

Tuberculosis (TB) and sepsis represent two archetypal forms of systemic infection. TB, caused by *Mycobacterium tuberculosis*, primarily manifests as pulmonary infection but can disseminate hematogenously, establishing extrapulmonary foci in organs such as bones and meninges. Its chronic, persistent nature constitutes a formidable global public health burden. Sepsis arises from the hematogenous invasion, proliferation, and toxin release of pathogens. Its clinical course can rapidly progress to systemic inflammatory response syndrome (SIRS), septic shock, and multiple organ dysfunction syndrome (MODS), culminating in significant mortality. While antimicrobial therapy remains the cornerstone of management, the emergence of resistant strains poses escalating challenges to conventional treatment paradigms. Critically, the dysregulated inflammatory response triggered by systemic infection is frequently the principal contributor to mortality, underscoring immunomodulation as a key research focus. Nanozymes exhibit a unique antimicrobial mechanism coupled with the capacity to scavenge excessive ROS, thereby exerting immunomodulatory effects. The rational design and application of nanozymes thus represents a promising therapeutic avenue for eradicating invading pathogens in systemic infections and mitigating associated severe inflammatory cascades.

**Multimodal Therapeutic Strategy** For sepsis induced by MDR bacteria, nanozymes can be integrated synergistically with diverse therapeutic modalities to enable multimodal intervention. For instance, Xuancheng Du's team engineered a biomimetic macrophage membrane-camouflaged nanozyme (designated MCeC@MΦ) for therapeutic application. This system employs mesoporous silica nanoparticles (MSNs) as the core, harboring CeO_2_ nanocatalysts and the photosensitizer Ce6. It achieves dual objectives of bacterial eradication and inflammation modulation by combining nanocatalytic therapy (NCT) with PDT to eliminate MDR pathogens, while leveraging the biomimetic membrane to neutralize endotoxins and pro-inflammatory cytokines. In a murine model of MDR *E. coli* bacteremia, MCeC@MΦ demonstrated multifaceted regulatory efficacy: significantly suppressing systemic hyperinflammation, reversing organ damage within 24 h, and elevating survival rates beyond 75 %. This platform thus offers a promising multimodal therapeutic paradigm for sepsis management(Fig. 9D) [131].

Nanomaterial-Based Catalytic Diagnostics and Targeted Intracellular Pathogen Strategies for Tuberculosis: *Mycobacterium tuberculosis* (Mtb), the causative agent of tuberculosis (TB), typically presents with minimal early-stage symptoms. Conventional diagnostic methods face limitations: sputum smear microscopy exhibits low sensitivity for smear-negative TB, while culture, despite higher sensitivity, is time-consuming. Although novel diagnostic technologies have emerged in recent years, their implementation in resource-limited settings remains challenging. Consequently, the development of efficient, rapid diagnostic assays independent of stringent laboratory infrastructure is critical for TB control. To address this, a specific Mtb detection platform was developed utilizing palladium-platinum bimetallic nanoparticles (Pd@Pt NPs) integrated with paper-based analytical devices and DNA hybridization techniques. This approach circumvents the laboratory dependency of conventional methods, offering potential for rapid point-of-care diagnosis in resource-poor regions [27].

Currently, first-line TB chemotherapy relies on a multidrug regimen of isoniazid (INH), rifampicin and its derivatives, pyrazinamide (PZA), and ethambutol (EMB). However, therapeutic efficacy is severely hampered by intrinsic physiological barriers: Mtb resides intracellularly within macrophages and induces granulomatous lesions at infection sites, substantially restricting drug bioavailability. This necessitates prolonged treatment durations with high drug doses. Inappropriate drug usage, compounded by these barriers, drives the emergence of drug-resistant strains, rendering MDR and extensively drug-resistant (XDR) TB increasingly formidable challenges. Nanozymes, distinguished by their potent antimicrobial activity and nanocarrier properties, hold promise for overcoming these therapeutic hurdles. Yet, research on nanozyme applications specifically targeting Mtb infection remains nascent.

Notably, a novel theranostic platform based on iron tetraphenylporphyrin (FeTPP) nanozyme-functionalized gold nanoparticles (FeTPP-AuNPs) has been reported. This system employs a bioorthogonal catalytic mechanism to selectively activate antibiotic prodrugs within macrophages, effectively eliminating intracellular pathogens such as Salmonella and Mtb. Such targeted catalytic strategies against intracellular pathogens provide a novel approach for treating diseases like typhoid fever and tuberculosis [28].

### 5.3 Specific infection models

**Fig. 10:**
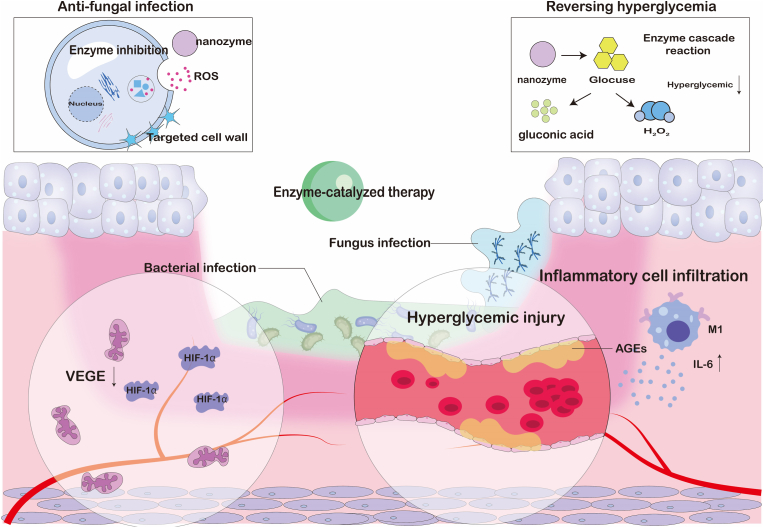
Schematic: nanozymes regulate the infection microenvironment—quelling inflammatory storms, relieving hypoxia, and reversing hyperglycemia.

Some infected areas often have complex physiological environments, such as hypoxia, hyperglycemia, persistent inflammatory storms, and fungal infections. Nanozymes are designed with "environmental reverse utilization" as the core, transforming hyperglycemia, hypoxia, and persistent inflammation at the site of infection from therapeutic barriers to bactericidal driving forces. In the face of hyperglycemia, the nanozyme loaded with GOx first catalyzes the conversion of glucose into gluconic acid and H_2_O_2_ within the lesion, cutting off the pathogen's carbon source and providing a substrate for its own POD-like activity, forming a self-circulating ROS amplification loop that causes cascade oxidative damage to drug-resistant and eukaryotic bacteria. At the same time, glucose consumption can reduce local osmotic pressure and alleviate hypertonic inflammation. In response to hypoxia, nanozymes can simulate cellular oxidases and convert endogenous O_2_^−^ and H_2_O_2_ into highly toxic •OH even under extremely low oxygen tension. They also catalyze the dismutation of H_2_O_2_ to release O_2_, locally supplementing oxygen to destroy anaerobic bacteria's hypoxia protection and restore immune cell respiratory burst. For persistent inflammation, the SOD/CAT like activity of nanozymes can instantly eliminate excessive ROS(Fig. 10).

#### 5.3.1 Diabetic infection model

Diabetic wound infections, with diabetic foot ulcers (DFUs) representing a particularly severe manifestation, pose formidable clinical challenges due to pathologically sustained hyperglycemia. This altered microenvironment fosters pathogenic colonization while concurrently compromising innate immune defenses, evidenced by impaired neutrophil chemotaxis/phagocytosis, diminished macrophage bactericidal activity, and consequent immune surveillance failure. Furthermore, hyperglycemia-induced mitochondrial dysfunction and persistent oxidative stress critically disrupt regenerative cascades, impeding wound closure. Conventional antibiotic therapies remain limited by emerging drug resistance, their narrow bactericidal focus failing to remodel the pathological niche, and potential immunosuppressive side effects. Nanozyme-based interventions capitalize on dynamically tunable enzymatic activities (e.g., ROS regulation, antioxidant mimicry) and inherent multifunctional integration, thereby offering promising alternatives that simultaneously target bacterial eradication, inflammation resolution, and tissue regeneration within the complex diabetic wound milieu.

**Fig. 11:**
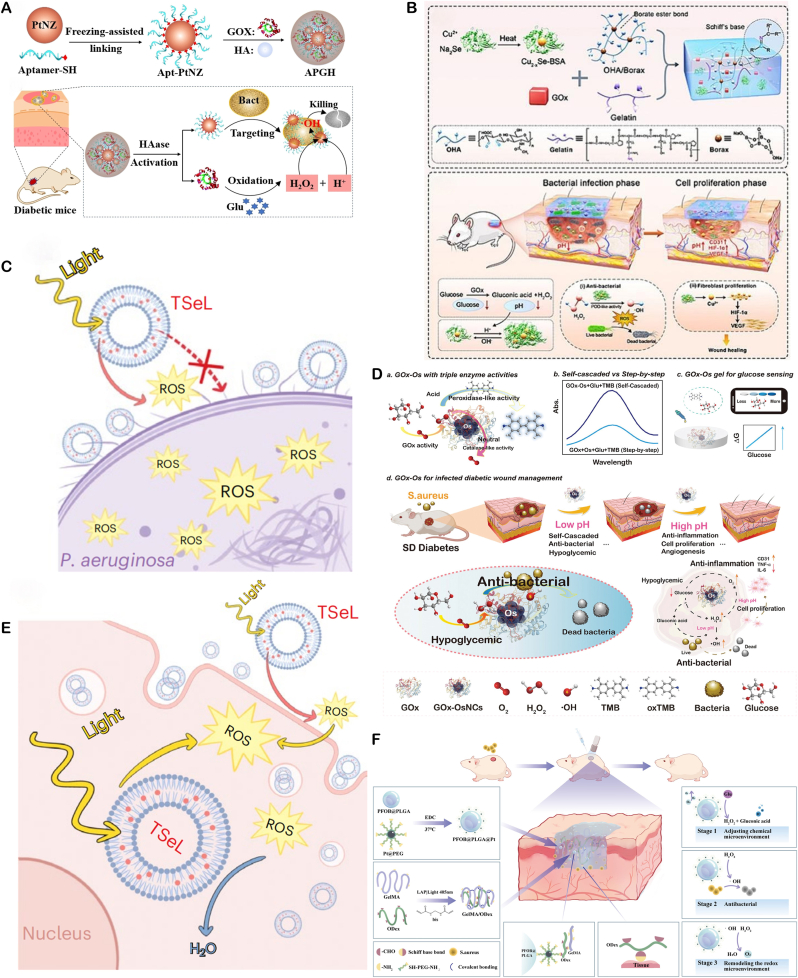
(**A**) Schematic of APGH nanozyme capsule fabrication—aptamer-Pt nanozymes, GOx and HA co-assembled—and its wound-infection response: selective bacterial binding activates a switch that drives in-situ COH generation on pathogen surfaces for chemodynamic sterilization. Copyright 2021 Wiley-VCH GmbH. (**B**) Schematic of OBG@CG: one-pot synthesis of OHA/borax-gelatin hydrogel embedding Cu_2_–xSe-BSA-GOx drives diabetic-wound healing via glucose depletion, sustained H_2_O_2_ supply, and cascade ROS-catalytic sterilization coupled with pro-angiogenic and anti-inflammatory modulation. Copyright 2024 Elsevier B.V. (**C, E**) Schematic of TSeL: co-localization and dual mechanisms—antibacterial action against bacteria, antioxidative protection in eukaryotes. Copyright 2025 Springer Nature Limited. (**D**) Schematic: triple-enzyme GOx-OsNCs (POD-, GOx-, CAT-like) form a self-cascaded glucose sensor outperforming stepwise GOx + OsNCs, are integrated into hydrogels for glucose detection, and deployed for infected diabetic wound therapy. Copyright 1999–2025 John Wiley & Sons, Inc or related companies. (**F**) Schematic of PFOB@PLGA@Pt/GelMA/ODex double-network nanohybrid hydrogel: multienzyme-mimetic sterilization and microenvironment remodeling with strong tissue adhesion accelerate diabetic wound healing. Copyright 2023 American Chemical Society.

**Multienzyme Cascade Reversal of Hyperglycemic Conditions:** The hyperglycemic microenvironment presents a primary challenge for treating infections in diabetic contexts. To circumvent this limitation, numerous researchers have employed strategies integrating nanozymes with GOx. This approach externally depletes excess glucose, alleviating hyperglycemic stress, while concurrently generating gluconic acid and hydrogen peroxide (H_2_O_2_) as catalytic products. These products establish favorable pH conditions and supply requisite substrates for subsequent enzymatic reactions. For instance, the APGH nanocapsule system (Aptamer-Pt/GOx@HA), developed by Lifang Chen et al. achieves precise therapeutic intervention through a pathogen-responsive mechanism. This system comprises an aptamer-modified platinum nanozyme, GOx, and hyaluronic acid (HA). Its innovations encompass: (1) Activation triggered by hyaluronidase secreted from pathogenic bacteria; (2) Enhanced bacterial surface interaction mediated by aptamer-targeted recognition; (3) In situ generation of H_2_O_2_ and localized pH reduction via glucose oxidation, synergizing with the POD-like activity of the platinum nanozyme to produce hydroxyl radicals. This design effectively addresses the constraints of insufficient endogenous H_2_O_2_ concentration and unfavorable pH in physiological settings, demonstrating marked antibacterial efficacy in both in vitro and animal models (Fig. 11A) [111]. Similarly, the Fe_2_(MoO_4_)_3_ composite system engineered by Yanfang Zhang et al. modulates the microenvironment through endogenous glucose metabolism, thereby circumventing the adverse effects associated with exogenous H_2_O_2_ supplementation while simultaneously overcoming pH restrictions [112].

**Intelligent Modulation of Oxidative Stress Homeostasis:** Excessive oxidative stress constitutes a primary impediment to wound healing. While elevated ROS production facilitates bacterial eradication, persistent oxidative stress detrimentally impacts tissue repair. Consequently, precise modulation of ROS levels is critical for effective wound regeneration. The dual-dynamic gel system OBG@CG, engineered by Sicheng Jiang et al. exhibits pH-responsive characteristics. Its innovative features include: (1) Acid-triggered self-assembly and activation of Cu_2-_xSe-BSA nanozymes, generating hydroxyl radicals for biofilm disruption; (2) Termination of ROS production upon pH elevation during later healing stages, switching to Cu^2+^ release to facilitate collagen synthesis; (3) Compromise of bacterial defense systems via interference with thiol metabolism and transporter activity, enabling a multifaceted antibacterial strategy (Fig. 11B) [113]. This dynamic regulation mechanism effectively adapts to microenvironmental shifts across distinct diabetic wound healing phases. Similarly, the GOx-OsNCs nanozyme system developed by Shaobin He et al. demonstrates pH-responsive dual-modal catalytic activity: under acidic conditions, it mediates bactericidal effects through a GOx-POD cascade reaction, while activating CAT-like activity to scavenge excess ROS under neutral/alkaline conditions. This intelligent switching mechanism concurrently addresses infection control and oxidative stress regulation (Fig. 11D) [114].

**Reprogramming the Immune Microenvironment to Resolve Chronic Inflammation:** The PFOB@PLGA@Pt nanohybrid hydrogel system enables multi-stage therapeutic intervention through the following innovations: (1) Perfluorooctyl bromide (PFOB)-mediated amelioration of wound hypoxia; (2) Self-regulating catalytic equilibrium for ROS generation and elimination via pH-responsive modulation; (3) Glucose metabolism orchestration through GOx-like activity coupled with SOD/CAT-mimetic functions to accelerate inflammation resolution(Fig. 11F) [115]. Concurrently, the Janus liposomal enzyme system (TSeL) achieves synergistic therapy via: (1) Photosensitizer-triggered ROS burst enabling eradication of drug-resistant pathogens (e.g., MRSA); (2) Selenium-based nanozyme-mediated scavenging of excess ROS to restore redox homeostasis; (3) Macrophage polarization reprogramming that potentiates tissue regeneration. This integrated platform achieved complete bacterial eradication and substantial epithelial regeneration in animal models, effectively addressing the dual challenges of infection control and healing promotion(Fig. 11C–E) [116].

Therapeutic Challenges in Diabetic Infections: Key impediments include dysregulated glucose metabolism, vasculopathy, immune imbalance perpetuating chronic inflammation, and recalcitrant bacterial colonization. Current research paradigms demonstrate that nanozyme-based strategies are progressively addressing these complications through: (1) Precision microenvironment-responsive design; (2) Synergistic orchestration of multienzyme activities; (3) Integration of dual therapeutic-reparative functionalities. These advances are overcoming inherent limitations of conventional therapies. Future investigations should prioritize: (1) Mechanistic interrogation of complex biointerfaces; (2) Comprehensive long-term biosafety assessment; (3) Development of patient-specific treatment regimens to accelerate clinical translation.

#### 5.3.2 Chronic refractory wound model

Persistent wounds, representing severe sequelae of diabetes, burns, and other pathologies, exhibit impaired healing primarily due to bacterially-driven vicious cycles. Pathogenic colonization establishes resilient biofilm barriers that perpetuate localized inflammatory responses and suppurative infections, potentially progressing to systemic manifestations including sepsis. This cascade exacerbates tissue destruction (increasing limb amputation risk) while concurrently disrupting the immune microenvironment to impede reparative processes. Recent advances confront this self-perpetuating pathological triad—antimicrobial resistance, microenvironmental dysregulation, and compromised tissue regeneration—through nanozymes with multienzyme-mimetic activities. These catalysts offer innovative therapeutic paradigms by leveraging unique antibacterial mechanisms for chronic wound management.

**Fig. 12:**
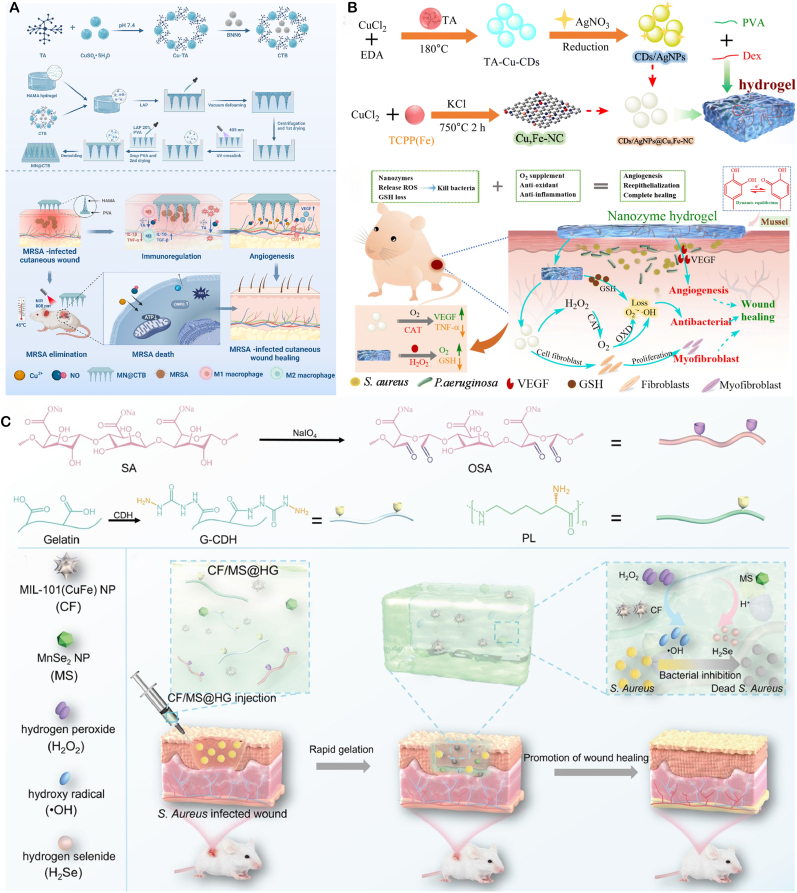
(**A**) Schematic of MN@CTB microneedle fabrication and synergistic therapy of MRSA-infected wounds Copyright 2025 Wiley-VCH GmbH. (**B**) hydrogel preparation and wound-healing mechanism. Copyright The Royal Society of Chemistry 2023. (**C**) Schematic of OSA/G-CDH/PL synthesis and CF/MS@HG: injectable rapid gelation, antibacterial action, and accelerated wound repair. Copyright Science China Press 2024.

**Intelligen Responsive Nanozyme Dressings:** The injectable microenvironment-responsive hydrogel (CF/MS@HG) demonstrates significant translational potential. This system achieves in situ gelation through Schiff-base crosslinking between oxidized alginate, aminated gelatin, and polylysine. Synergistic encapsulation of MIL-101(CuFe) nanoparticles (CF NPs) and manganese selenide nanoparticles (MnSe_2_ NPs, MS NPs) establishes dual antibacterial mechanisms: (1) CF NPs exhibit POD-mimetic activity, catalyzing H_2_O_2_ conversion into hydroxyl radicals (•OH) within wound microenvironments to disrupt bacterial integrity via oxidative damage; (2) Acid-triggered release of hydrogen selenide (H_2_Se) from MS NPs suppresses proliferation through metabolic interference. In vitro analyses confirm superb tissue adhesion and self-healing properties, while polylysine potentiates antibacterial efficacy. Animal models reveal not only favorable biosafety profiles but also markedly accelerated wound regeneration. This work establishes a pivotal paradigm for developing intelligent responsive biomaterials (Fig. 12C) [117].

**Multifunctional Nanozymes for Multidimensional Wound Management:** Chronic wounds present multifaceted pathological hallmarks encompassing antimicrobial resistance, biofilm formation, persistent oxidative stress, dysregulated inflammation, and impaired regenerative capacity. Single-activity nanozymes exhibit limited efficacy against such complexity. Developing integrated multifunctional nanozyme systems is therefore critical for addressing the intricate pathology of refractory wounds.

The Cu-HMCe nanozyme achieves breakthrough progress by concurrently addressing three core pathological mechanisms: bacterial colonization, oxidative stress, and vascular regeneration through Cu^2+^-mediated polypharmacology. Specifically: (1) Cu^2+^ exerts potent bactericidal effects against *S. aureus* and *E. coli* via membrane disruption, enzymatic interference, and ROS induction; (2) Its antioxidant-mimetic activity scavenges excess ROS, mitigating oxidative damage; (3) Critically, copper activates endothelial migration/proliferation pathways to drive neovascularization and microcirculatory improvement. This tripartite therapeutic modality pioneers new avenues for chronic wound intervention [118]. A Cu^2+^-tannic acid nanozyme (CTB) loaded with the nitric oxide (NO) prodrug BNN6 was developed and integrated into methacrylated hyaluronic acid microneedles (MN@CTB). Under 808 nm near-infrared irradiation, the MN@CTB system concurrently exhibited photothermal effects and controlled NO release, leading to highly efficient synergistic eradication of MRSA, with an in vitro antibacterial rate exceeding 94 % and an 89 % reduction in bacterial load in vivo. Furthermore, the antioxidant activity of CTB suppressed reactive oxygen species (ROS) accumulation via the Nrf-2/HO-1 pathway, downregulated NF-κB-mediated inflammatory responses, and promoted macrophage polarization from the M1 to the M2 phenotype. The released NO additionally activated the sGC/PKG signaling pathway, upregulating VEGF and TGF-β expression, which significantly enhanced angiogenesis and collagen deposition. As a result, complete healing of MRSA-infected wounds in mice was achieved within 14 days(Fig. 12A) [119].

Complementing this, the multifunctional biomimetic nanozyme hydrogel engineered by Yang Dezhi's team (Kunming University of Science and Technology) extends the frontier through synergistic integration of tea polyphenol-derived copper-carbon dots/reduced silver nanoparticles (CDs/AgNPs) and copper/iron-nitrogen-doped carbon (Cu,Fe-NC). Key innovations include: (1) Bacterial antioxidant defense collapse via GSH depletion mimicry coupled with precision ROS generation; (2) Catechol-mediated surface functionality enabling mussel-inspired adhesion for stable wound coverage while sustaining antibacterial activity through dynamic redox homeostasis. In vivo validation confirms effective bacterial suppression, inflammatory microenvironment modulation, and significantly accelerated re-epithelialization. This biomimetic design establishes a robust theoretical framework for next-generation wound therapeutics(Fig. 12B) [120].

These advances demonstrate that multifunctional nanozyme-hydrogel systems, integrating microenvironmental responsiveness, multimodal antimicrobial mechanisms, and pro-regenerative functionality, are driving the evolution of chronic wound management toward precision-controlled, adaptive therapeutic strategies. Future investigations must prioritize: (i) comprehensive long-term biosafety assessment, (ii) development of scalable manufacturing protocols, and (iii) translation to real-world clinical scenarios with complex comorbidities.

#### 5.3.3 Fungal infection model

Fungal infection, as another important type of pathogenic infection after bacterial infection, faces severe challenges in its treatment. Unlike bacterial infections, fungal infections are more likely to occur on the skin and mucous membranes (such as hand and foot moss, fungal keratitis, vaginitis). At present, there are many difficulties in the treatment of fungal infections: the skin barrier hinders drug penetration, and the drug utilization for deep fungal infections (such as fungal keratitis) is severely insufficient; fungi, such as Candida albicans, are prone to developing resistance to azole drugs, but the mechanism is different from that of bacteria. They mainly adapt to resistance through biofilm and metabolism; Fungi are also eukaryotic organisms, and most mainstream antifungal drugs have significant side effects on the human body. Nanozymes, with their unique catalytic activity and targeting ability, also have the potential to demonstrate significant potential in the field of antifungal treatment. So far, research cases have revealed innovative breakthroughs in antifungal mechanisms and therapeutic effects of different nanozyme systems [121].

**ROS mediated fungal cell damage** The unique ROS generation mechanism of nanozymes enables them to also have a killing effect on fungi. Research has shown that cerium based metal organic framework nanozymes (Ce MOF, AU-1) exhibit broad-spectrum antifungal properties. Through systematic evaluation using colony forming units, dry mass method, soluble protein detection, and microscopic imaging, the results showed that the nanozyme exhibited inhibition efficiencies of 93.3 %–99.3 % against Aspergillus flavus, Aspergillus niger, Aspergillus terreus, Candida albicans, and Rhodotorula glutinis. Its efficient antimicrobial activity is attributed to the oxidative stress caused by the Ce^3+^/Ce^4+^ redox cycle, which damages the integrity of fungal cell walls and interferes with metabolic processes [122].

**Fig. 13:**
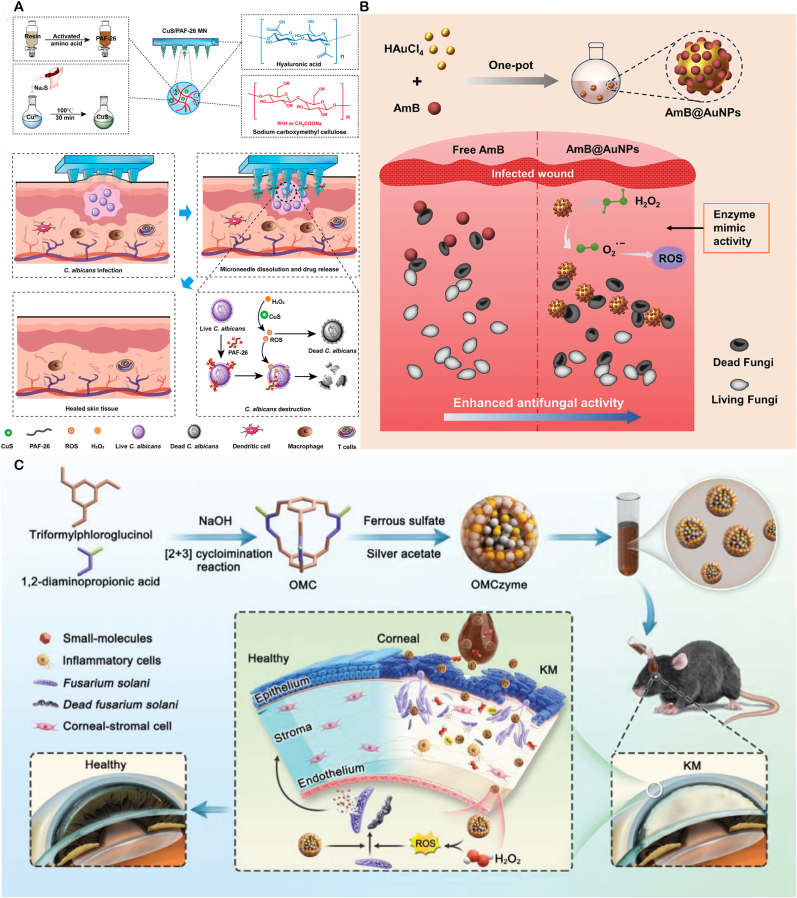
(**A) Schematic** of CuS/PAF-26 MN: molded by PDMS, the microneedles adhere, dissolve, and continuously release CuS nanoclusters (ROS via H_2_O_2_ catalysis) and membrane-disrupting PAF-26 for synergistic fungal eradication in DCFI. Copyright 2023 American Chemical Society. (**C**) Schematic of AmB@AuNP synthesis and its antifungal wound-healing mechanism. Copyright 2024 Wiley-VCH GmbH. (**D**) Schematic of ionic OMCzyme synthesis for keratomycosis therapy. Copyright 2024 Wiley-VCH GmbH.

**Synergistic effect of nanozyme combined with antifungal drugs** Nanozyme combined with traditional antifungal drugs can reduce the dosage of antifungal drugs, decrease side effects, and effectively control the development of fungal resistance. Amphotericin B-modified gold nanoparticles designed by Chunmei Jiang's team (AmB@AuNPs) The dual mechanism of drug enhancement and catalytic killing has been achieved. This system enhances the antifungal effect through the following innovative points: (1) AmB modified on the surface of gold nanoparticles enhances the binding specificity of ergosterol to fungal cell walls; (2) POD-like activity catalyzes the conversion of H_2_O_2_ into highly toxic hydroxyl radicals (•OH), synergistically disrupting cell membrane structure. Experiments have shown that compared to free amphotericin B, AmB@AuNPs The MIC of Candida albicans and *Saccharomyces cerevisiae* were reduced by 1.6 times and 50 times respectively, significantly accelerating the healing of fungal infection wounds and demonstrating good biocompatibility (Fig. 13B) [123].

**Enhancing drug delivery to overcome physiological barriers** Xia's research team has developed a biodegradable microneedle patch (Cus/PAF-26MN) fabricated from hyaluronic acid (HA) and sodium carboxymethyl cellulose (CMC Na) for the antibiotic-free treatment of deep cutaneous fungal infections (DCFI). The microneedle system enables the co-delivery of copper sulfide nanozymes (CuS NE) and the antimicrobial peptide PAF-26. The CuS NE exhibits POD-like catalytic activity, generating ROS from endogenous H_2_O_2_ to exert fungicidal effects, while PAF-26 disrupts fungal membrane integrity. This dual mechanism of action demonstrates synergistic antifungal efficacy while minimizing the potential for resistance development(Fig. 13A) [124]. Due to its unique physiological barrier, antifungal drugs are difficult to penetrate deeply into the cornea and have extremely low drug utilization in corneal fungal diseases. The OMCzzyme nanozyme system breaks through the treatment bottleneck through multi-level design: (1) constructing a three-dimensional skeleton based on penetrating ion organic molecular cages (OMCs) to endow excellent corneal penetration; (2) Fe^2+^ coordination modification and silver nanoparticle deposition form dual active centers, possessing both POD-like activity and silver ion antimicrobial properties. In the Fusarium solani infection model, OMCzyme catalyzes the generation of ROS from H_2_O_2_ to disrupt fungal cell membranes, while silver nanoparticle deposition triggers membrane potential disruption, achieving a fungal clearance rate of 96.8 % and toxicity to human corneal epithelial cells less than 5 % (Fig. 13C) [125].

**Multi enzyme catalysis + physical destruction multi-mechanism synergistic effect:** IHIHICI heptapeptide self-assembled nanozyme opens up a new strategy for biomimetic catalysis and antifungal treatment. The innovation of this system is reflected in: (1) the optimized design of β-helical nanotube structure through computer simulation, achieving efficient binding with fungal cell wall mannan; (2) Nickel ion coordination activates dual enzyme activity-phospholipase C (PLC) degrades the cell membrane phospholipid layer, and POD-like activity catalyzes lipid peroxidation reaction. In terms of antifungal activity, IHIHICI nanozymes exhibit highly efficient antifungal activity, particularly against Candida albicans. Experimental results have shown that a large number of Ni-IH-7 twined with Candida albicans to form a cocoon like structure, resulting in the destruction of cell wall integrity, the expansion of nuclear area and the disappearance of subcellular organelles such as mitochondria. The nanozyme can remove more than 90 % of Candida within 10 min. Its mechanism of action involves the change of cell membrane permeability (increased by 3.2 times), depolarization of membrane potential (decreased by 78 % in ΔΨ) and activation of iron death pathway, providing a multi-target intervention scheme for antifungal treatment [22].

The current research trend indicates that the application of nanozymes in the field of antifungal treatment is shifting from a single killing mechanism to multi-mode synergistic therapy. By combining biomimetic catalysis, targeted modification, and intelligent response design, it is expected to break through the resistance limitations of traditional antifungal drugs and provide a new approach for the treatment of deep fungal infections in the future.

#### 5.3.4 Antiviral and antiparasitic strategies of nanozymes

Nanozymes are a type of nanomaterial with enzyme like catalytic activity, which not only can combat bacteria, but also demonstrate unique application potential in the field of viral and parasitic infections. Its value lies in providing a new strategy that is efficient, broad-spectrum, and less likely to induce drug resistance against pathogens that are prone to traditional drug failure.nanozymes exert their effects by directly disrupting the structure of pathogens and regulating the host immune environment, and their strategies are very flexible.

Multiple strategies for combating viruses

Nanozyme antiviral effects are mainly achieved by directly disrupting the virus structure and regulating the host immune response. (1) Direct attack: Many viruses (such as influenza and coronavirus) have a lipid bilayer outer membrane. Iron based and manganese based nanozymes can simulate oxidases and catalyze lipid peroxidation of viral envelope, leading to membrane rupture and loss of embedded proteins (such as hemagglutinin), thereby rendering the virus unable to infect. This effect targets the conserved physical structure of the virus and therefore has a broad spectrum [132]. (2) Immune regulation: Viral infections often cause excessive oxidative stress in the body. Cerium based nanozymes can simulate SOD and CAT, eliminate excess ROS, alleviate inflammatory reactions and tissue damage, and help the body recover. For example, in a viral pneumonia model, inhalable cerium based nanozymes can effectively alleviate lung inflammation [133].

Collaborative mechanism for combating parasites

Nanozymes also demonstrate unique advantages in combating parasitic infections. Ferritin nanozyme is an intelligent nanomaterial constructed through biomimetic strategies, which uses the endogenous heavy chain ferritin (HFn) in the human body as its shell, endowing it with the unique ability to target transferrin receptor 1 (TfR1) in endothelial cells of the blood-brain barrier, and encapsulating a core of Fe_3_O_4_ nanoparticles with CAT-like activity inside. In the treatment of central nervous system infections such as cerebral malaria, it plays a dual key role: on the one hand, it efficiently removes excess ROS caused by infection, protecting the integrity of the blood-brain barrier like a "molecular fire extinguisher", reducing fatal brain edema and neuroinflammation, and directly protecting the host; On the other hand, it can polarize liver macrophages into M1 phenotype, enhancing their ability to clear parasites. Of particular importance is that when combined with the traditional antimalarial drug artemether, ferritin nanozymes form a synergistic therapy of "insecticidal" and "life-saving" artemether rapidly kills parasites, while nanozymes focus on alleviating pathological damage and improving neurological symptoms, thereby significantly improving efficacy and demonstrating enormous clinical potential as adjuvant therapy [134].

The discussed investigation into nanozyme design and development for specific pathogenic infections reveals that research in this field is increasingly evolving to address targeted clinical scenarios. While substantial breakthroughs have been achieved in preclinical studies, the translation of these findings from laboratory settings to clinical application continues to present substantial hurdles.

## 6 Navigating the preclinical landscape of nanozymes: advances and barriers to clinical translation

### 6.1 Nanozymes in antimicrobial: from bench to Bedsid–Trailblazing advances and translational milestones

#### 6.1.1 Key breakthroughs and clinical translation stage

Currently, most nanozyme research aimed at replacing clinical antibiotics for human disease treatment remains in the preclinical stage, with very few advancing to human clinical trials. Nevertheless, significant technological breakthroughs have been achieved in preclinical studies, particularly in the areas of combination therapy, intelligent responsiveness, and rational design.

##### 6.1.1.1 Multi-enzyme Cascade Catalysis and combination therapy

Single-mechanism therapies often yield limited efficacy. Contemporary designs increasingly favor the construction of synergistic "1 + 1>2" therapeutic systems, enabling nanozymes to surpass traditional antimicrobial agents by not only targeting bacteria but also promoting disease healing [135]. For instance, integrating the antioxidant capacity of nanozymes with the immunomodulatory functions of probiotics [136], or combining catalytic therapy with immune cell regulation, allows for multi-mechanism, multi-target interventions against complex diseases.

##### 6.1.1.2 Development of smart-responsive nanozymes

Early-generation nanozymes functioned more like systemic "wanderers", whereas current research focuses on transforming them into "targeted warheads". Through techniques such as ligand modification or biomembrane coating to enhance specific recognition, or by leveraging unique chemical features of bacterial infection sites (e.g., pH, oxygen levels) and external stimuli (e.g., lasers) for remote and precise activation, researchers aim to achieve highly efficient localized action while minimizing systemic side effects. Moreover, smart-responsive nanozymes can "intelligently" initiate and even switch therapeutic logic based on changes in the pathological microenvironment. For example, a glucose-responsive nanozyme-hydrogel platform developed by a team at Shanghai Jiao Tong University autonomously triggers a cascade reaction under high-glucose conditions: it consumes glucose to generate hydrogen peroxide, which is then catalyzed in the acidic microenvironment to produce •OH for bacterial eradication, while simultaneously releasing insulin to assist in glucose reduction. Such feedback-regulated mechanisms enable autonomous functional switching according to microenvironmental changes, embodying true "intelligent" diagnosis and treatment [137]. Additionally, smart-responsive nanozymes can achieve on-demand release through environmental triggers, facilitating controlled and sustained release that greatly mitigates potential toxic side effects associated with dosage inaccuracies [101,138].

##### 6.1.1.3 AI-assisted rational design

Traditional nanozyme development largely relied on trial-and-error experimentation. With the advancement of artificial intelligence, the field has undergone a transformative shift toward efficient "rational design". By training models on high-throughput experimental data, it is now possible to predict the enzyme-like activities of nanomaterials (such as metal oxides and carbon-based materials). Furthermore, large-scale material science models are being utilized to screen potential nanozyme compositions [24,25,139].

#### 6.1.2 Clinical translation stage

To date, research on nanozymes remains in the preclinical stage, with the majority of studies confined to animal models and only a very limited number conducted in human models. Large-scale clinical applications have not yet been realized. Nevertheless, several advances demonstrate considerable translational promise, with multiple projects approaching a "tipping point" toward clinical translation [140,141]. Some representative candidates are advancing from Pre-IND/IND submission toward early-phase human trials, positioning them in a "quasi-clinical" stage.

##### 6.1.2.1 Standardizing nanozymes for clinical translation

In 2024, Yan's group proposed a comprehensive design framework for the in vivo application of nanozymes in Nature Reviews Bioengineering. They emphasized the need to enhance pharmaceutical suitability by improving structural uniformity, optimizing pharmacokinetics, assessing immunotoxicity, and formalizing standards for Chemistry, Manufacturing, and Controls (CMC). Collaborative efforts are underway within China's scientific community to establish quality control standards and regulatory frameworks for nanozymes. These initiatives aim to lay the foundation for IND submissions and eventual clinical translation [142].

##### 6.1.2.2 Nanozyme activity rediscovery: A promising translational bridge

The rediscovery of nanozyme activity in approved drugs offers a promising translational bridge. Ferumoxytol (brand name Feraheme), an ultra-small superparamagnetic iron oxide nanoparticle, is FDA-approved for intravenous iron supplementation in chronic kidney disease (CKD) patients. Recent studies have revealed that it also exhibits catalase (CAT)-like activity, enabling reactive oxygen species (ROS) scavenging and conferring functions typically associated with nanozymes. While its approved indication does not rely on nanozyme mechanisms, its established safety profile in humans provides critical reference points for the future design and translation of nanozyme-based therapeutics [143].

##### 6.1.2.3 Several nanozyme candidates have completed preclinical studies and are undergoing or preparing for clinical trial applications

Several nanozyme candidates have completed preclinical studies and are undergoing or preparing for clinical trial applications. South Korea's Cenyx Biotech has developed four cerium oxide-based nanozyme pipelines. CX213, targeting subarachnoid hemorrhage and acute liver failure, has completed preclinical studies and is advancing toward clinical trials. CX301, designed for refractory stroke and acute respiratory distress syndrome, is expected to submit an IND application in 2025. Nanozyme-D (topical, for atopic dermatitis) and Nanozyme-E (oral, for ulcerative colitis/Crohn's disease) have demonstrated efficacy in animal models and are in preparation for IND submission. These candidates represent the most clinically advanced systemic nanozyme drugs globally and are supported by South Korean governmental and national research funds, enhancing their translational prospects.

Despite these projects approaching the clinical threshold, only two studies—titled "Programmed Therapy of Diabetic Foot Ulcers Using a Dual-Responsive Multifunctional Hydrogel Driven by Diatomic Nanoenzymes" and "Clinical observation on the treatment of chronic actinic dermatitis and solar dermatitis with C-dot nanoe"—has been registered on the Chinese Clinical Trial Registry (ChiCTR), Registration number:(ChiCTR2500107916) (ChiCTR2200063015). Globally, only an iron oxide nanozyme project from China has formally entered human studies. Cenyx's CX213 and CX301 are projected to commence Phase I trials in 2025–2026, positioning them as the most translationally promising systemic nanozyme therapeutics. Multiple research teams in China are also advancing nanozyme applications in ophthalmology, gastroenterology, and neurology, currently transitioning from animal studies toward clinical evaluation. Collectively, these developments indicate that the field of nanozymes is in a transitional phase from preclinical to clinical research, yet a full-fledged "clinical stage" has not yet been comprehensively initiated.

### 6.2 Challenges towards clinical practice

Despite the considerable promise and advantages of nanozymes, several critical challenges must be addressed before their successful translation into clinical practice: 1)Biosafety and Toxicity Concerns. This remains the primary barrier to clinical application. Our current understanding of the long-term biosafety of nanozymes within the complex human physiological environment remains incomplete. Key issues include whether nanozymes accumulate persistently in the body and induce chronic damage to organs such as the liver and kidneys—questions that necessitate systematic long-term investigation. The degradation pathways, metabolic fate, and the capacity of the body to efficiently and safely clear different types of nanozymes are central to safety evaluation. Certain inorganic nanozymes may resist natural clearance mechanisms. Moreover, their recognition by the immune system as foreign entities could trigger unintended immune responses or inflammatory storms, potentially exacerbating tissue injury. Current biosafety evaluation of nanozymes primarily involves cytotoxicity assays to assess their impact on various cell lines such as hemolysis tests and MTT assays [144,145] or murine models to examine in vivo distribution, metabolism, and long-term toxicity, including histopathological analysis of major organs following nanozyme administration. Furthermore, the development of composite nanozyme systems, which integrate the advantages of different materials, has been shown to enhance both antimicrobial performance and biosafety [[146], [147], [148]]. Numerous studies have confirmed that properly engineered nanozymes exhibit favorable biocompatibility without inducing significant cytotoxicity or tissue damage. 2) Catalytic Efficiency and Selectivity. Although many nanozymes have demonstrated high ROS generation efficiency and significant therapeutic outcomes in preclinical animal models, there is currently no clinical data to confirm whether they can maintain catalytic activity and stability in the complex and variable milieu of human disease. 3) Scalability from Laboratory to Industrial Production. Transitioning from gram-scale synthesis in the laboratory to kilogram- or even ton-scale industrial production represents a major hurdle. Presently, the variety of high-performance nanozyme materials—particularly those responsive to stimuli such as NIR-II light for deep-tissue therapy—remains limited. Scaling up production while ensuring batch-to-batch consistency in size, morphology, surface chemistry, and catalytic activity is a fundamental challenge. Even minor deviations may lead to significant variations in efficacy and safety. Furthermore, designing nanozymes that integrate multiple functions—such as bacterial membrane adhesion, biofilm penetration, and ROS generation—to tackle resilient infections like biofilms introduces considerable complexity. The industrial feasibility and cost-effectiveness of such multifunctional integrated systems remain to be thoroughly evaluated. 4) Standardized Regulatory and Evaluation Frameworks. As a novel class of antimicrobial agents, nanozymes lack a mature and uniform regulatory pathway. There is an absence of well-established, standardized evaluation criteria or technical guidelines concerning their safety, efficacy, and quality controllability. This regulatory ambiguity introduces uncertainty into the approval process for clinical trials.

## 7 Conclusion and outlook

Nanozymes, as artificial mimics that combine the catalytic activity of natural enzymes with the unique properties of nanomaterials, hold distinct advantages in combating pathogenic microbial infections, particularly those caused by drug-resistant bacteria. Their ability to circumvent bacterial resistance stems from mechanisms such as ROS generation, immune modulation, and physical disruption—modes of action fundamentally different from those of conventional antibiotics. Moreover, nanozymes exhibit remarkable potential for synergistic therapies. Their integration with physical antimicrobial approaches—such as photothermal/photodynamic therapy and sonodynamic therapy—as well as their use as targeted carriers for antibiotic delivery, helps mitigate antibiotic overuse and offers novel strategies to address the global crisis of AMR. This review comprehensively explores the applications of nanozymes in anti-infective therapy, with a specific focus on their promise in treating bacterial infections, which pose a major public health threat amid the escalating AMR challenge. We systematically examine antimicrobial nanozymes from three perspectives: classification, catalytic mechanisms, and antimicrobial modes of action, placing special emphasis on the enzyme-mimetic activities that underpin their antimicrobial efficacy. Given their unique mechanisms for countering drug-resistant pathogens, future development should prioritize the design of nanozyme systems tailored to clinically relevant infectious diseases, thereby facilitating their clinical translation and potential to replace conventional antimicrobial agents. Accordingly, we highlight applications of nanozymes in bacterial and fungal infection models, including diabetic wounds, chronic ulcers, oral infections, ocular inflammation, and biofilm-associated infections, where they have demonstrated excellent antimicrobial performance. However, research on nanozymes in the field of pathogenic infections must be further deepened and expanded.

(1) There remains a need to broaden investigations into nanozyme applications in clinically significant areas such as viral, parasitic, and fungal infections, tuberculosis, and systemic infections.
(2) AI is driving a paradigm shift in nanozyme research. Machine learning and graph neural networks enable high-accuracy activity prediction models based on physicochemical and atomic structural features; generative AI allows inverse design and multi-objective optimization for tailored catalytic performance and biocompatibility; multiscale simulations combined with active learning significantly expedite mechanistic studies and high-throughput screening. Furthermore, bio-inspired algorithms open new avenues for rational design by emulating natural enzyme active sites and evolutionary pathways. These integrated approaches are systematically advancing nanozyme development from empirical exploration toward programmable, automated design. Nevertheless, it must be recognized that AI-designed nanozymes still fall short of natural enzymes in structural complexity, particularly for multi-step catalytic reactions. Future efforts should focus on hybrid strategies that combine AI with traditional computational methods, seeking optimal trade-offs among catalytic efficiency, bacterial targeting specificity, biocompatibility, and production costs.
(3) Smart responsive nanozymes represent a concrete step toward precision medicine in anti-infective therapy. By incorporating physical (e.g., light, sound, magnetic), chemical (e.g., pH, ROS, glucose), and biological (e.g., enzyme, ATP) response modules, nanozymes can autonomously modulate their catalytic activity and antimicrobial behavior in response to specific biomarkers within the infection microenvironment. For instance, pH/ATP dual-responsive systems for gastrointestinal infections enable site-specific antimicrobial release by leveraging pH variations along the digestive tract and pathogen metabolic traits. Such spatiotemporally precise therapeutic strategies not only enhance treatment efficacy but also minimize systemic toxicity.
(4) Nanozymes demonstrate unique strengths in multimodal imaging-guided theranostics.The future experimental paradigm will naturally integrate diagnostic and therapeutic functions. Integrating complementary imaging modalities—such as the high spatial resolution of photoacoustic imaging, deep tissue penetration of magnetic resonance imaging, and high sensitivity of fluorescence imaging—can provide comprehensive information for precisely targeted antimicrobial therapy. For example, zinc–manganese ferrite nanoparticles developed by Shandong University successfully combine photoacoustic/MR dual-modality imaging with photothermal/catalytic synergistic therapy, offering a valuable model for precision antimicrobial applications. More advanced still are closed-loop theranostic systems, where real-time imaging feedback guides adjustable treatment parameters, enabling truly personalized and precise antimicrobial therapy. Imaging signals generated by nanozymes can reflect infection severity and intelligently regulate photothermal intensity or catalytic activity, maximizing efficacy while minimizing tissue damage.
(5) In terms of clinical translation, despite promising laboratory breakthroughs, the clinical application of nanozymes remains limited. Only a few nanozyme-based products for pathogen detection have entered the market, and their use remains constrained by industrial-scale manufacturing and quality control requirements. Most nanozyme research for clinical infections remains at the preclinical stage, underscoring the urgent need to address key challenges such as biosafety evaluation, material engineering optimization, and scalable production.

In summary, with the ongoing integration of AI-aided design, smart responsive systems, and multimodal imaging, nanozymes are evolving toward greater precision, efficacy, and safety. Future research should prioritize the development of adaptive nanozyme systems capable of dynamically tuning their catalytic behavior in response to real-time therapeutic needs; the construction of sophisticated multimodal imaging-guided theranostic platforms for truly individualized anti-infective treatments; and the establishment of standardized evaluation systems and scalable manufacturing processes to accelerate clinical translation. Nanozyme technology not only holds promise for mitigating the current AMR crisis but may also reshape the future landscape of anti-infective therapy. Through continued interdisciplinary collaboration, nanozymes are poised to offer innovative clinical solutions and make significant contributions to global public health in the near future.

## CRediT authorship contribution statement

**Shanshan Feng:** Writing – original draft. **Zhaoxun Wang:** Writing – review & editing. **Yibin Zhang:** Supervision. **Ling Mei:** Writing – review & editing, Supervision, Conceptualization. **Zhenxing Wang:** Writing – review & editing, Supervision.

## Declaration of competing interest

The authors declare that they have no known competing financial interests or personal relationships that could have appeared to influence the work reported in this paper.

## Data Availability

No data was used for the research described in the article.
